# Energy and Environment-Aware Path Planning in Wireless Sensor Networks with Mobile Sink

**DOI:** 10.3390/s22249789

**Published:** 2022-12-13

**Authors:** Fatma H. El-Fouly, Ahmed B. Altamimi, Rabie A. Ramadan

**Affiliations:** 1 Department of Communication and Computer Engineering, Higher Institute of Engineering, El-Shorouk Academy, El-Shorouk City 11837, Egypt; 2 Computer Engineering Department, College of Computer Science and Engineering, University of Ha’il, Ha’il 8650, Saudi Arabia; 3 Department of Computer Engineering, Faculty of Engineering, Cairo University, Giza 12613, Egypt

**Keywords:** path planning, sensor networks, energy, environmental, mobile sink, routing

## Abstract

With the advances in sensing technologies, sensor networks became the core of several different networks, including the Internet of Things (IoT) and drone networks. This led to the use of sensor networks in many critical applications including military, health care, and commercial applications. In addition, sensors might be mobile or stationary. Stationary sensors, once deployed, will not move; however, mobile nodes can move from one place to another. In most current applications, mobile sensors are used to collect data from stationary sensors. This raises many energy consumption challenges, including sensor networks’ energy consumption, urgent messages transfer for real-time analysis, and path planning. Moreover, sensors in sensor networks are usually exposed to environmental parameters and left unattended. These issues, up to our knowledge, are not deeply covered in the current research. This paper develops a complete framework to solve these challenges. It introduces novel path planning techniques considering areas’ priority, environmental parameters, and urgent messages. Consequently, a novel energy-efficient and reliable clustering algorithm is proposed considering the residual energy of the sensor nodes, the quality of wireless links, and the distance parameter representing the average intra-cluster distance. Moreover, it proposes a real-time, energy-efficient, reliable and environment-aware routing, taking into account the environmental data, link quality, delay, hop count, nodes’ residual energy, and load balancing. Furthermore, for the benefit of the sensor networks research community, all proposed algorithms are formed in integer linear programming (ILP) for optimal solutions. All proposed techniques are evaluated and compared to six recent algorithms. The results showed that the proposed framework outperforms the recent algorithms.

## 1. Introduction

A wireless sensor network (WSN) is a type of network with small, battery-powered sensor nodes that collect data from monitored environments. Such nodes are connected via wireless links to send their collected data to a sink node. However, one of the challenges of WSNs is energy consumption, particularly in large-scale areas with thousands of sensor nodes, since the main supply of energy in sensor nodes is batteries. At the same time, it is hard to recharge the batteries in some monitored areas, especially in hazardous or inaccessible areas. Thus, researchers’ primary considerations in this field are the nodes’ energy consumption and network lifetime [1,2].

Clustering conserves sensor node energy and improves network longevity. In clustering, the network is divided into groups. Every group has a head or leader. Cluster heads take sensor node data, aggregate it, and transfer it to the sink node directly or via other cluster heads [3]. Cluster heads may lose energy faster than conventional sensor nodes due to the extra load of receiving, aggregating, and transferring data to the sink node. Therefore, cluster heads must be energy-efficient because of transmission and reception operations. The death of these nodes in an area might cause network partitioning and data loss. Thus, proper selection of cluster heads conserves sensor node energy, prolonging network life. Several clustering approaches assign cluster heads based on the average distance to maximize cluster head lifespan in this setting. So, cluster heads are close member nodes. Certainly, if the cluster head dies, it may generate a serious energy imbalance in the network. To increase network lifetime, an energy-balanced clustering strategy is required [4,5]. It is obvious that high-quality, reliable wireless links enabling communication among sensor nodes would be required to successfully deliver the data packet from the member nodes to their cluster heads. However, due to wireless communication’s inherent nature in sensor networks, packet losses are quite inevitable. Wireless connections are prone to network disturbances due to some environmental parameters such as interference and fading. This would increase the retransmission possibility of lost packets and thus causes more energy consumption and more delivery delay. Nevertheless, most of the existing clustering techniques attempted to select cluster heads based on energy factors to improve network lifetime. Such approaches lack knowledge related to data transmission interruptions due to lossy links. This eventually affects the network throughput and lifetime. Therefore, the quality of wireless links is another factor that needs to be considered when designing the clustering technique for WSNs [6,7,8,9].

Although the clustering technique contributes to extending the network lifetime, there are still existing issues such as hotspot problems and connectivity. As the most consuming energy in sensor nodes comes from communication, the sensor nodes near to the static sink are at risk of dying more quickly than other nodes because they consume more energy by transmitting accumulative data to the static sink, causing hotspot problems. As a consequence, network lifetime decreases. Therefore, a mobile sink was proposed as one solution to the energy consumption issue in WSNs. The mobile sink may travel near the monitored area events through a planned path, which refers to scheduling the mobile sink’s movement and collecting the needed data from sensor nodes. Finding the ideal path for the mobile sink involves collecting data from sensor nodes [10]. Minimizing nodes’ distance from the mobile sink certainly conserves energy; it reduces multi-hop communication and addresses hotspot problems, extending network life.

The battery is the mobile sink’s primary power source in most applications. Thus, saving energy is crucial to a mobile sink’s lifespan. The mobile sink’s movement is a key source of energy consumption and costs. Most WSN path planning algorithms try to decrease energy usage to extend the lifetime of the mobile sink. It is important to design an energy-efficient path planning method [11].

The WSNs deployment environment may be heterogeneous. So, the monitored field is divided into small parts called zones, and a certain value is assigned to each zone indicating its priority (importance), namely, its zone priority [12]. The messages coming from sensor nodes located in the high-priority zones are considered urgent data. As urgent data are considered high-priority data, they must be transmitted immediately to the sink node. Suppose such messages occur in an exceptionally high-priority zone that the mobile sink has not visited yet or has visited already. In that case, they should be delivered promptly through a routing technique. Therefore, the mobile sink should move first to the high-priority zones to avoid excessive routing of urgent data. Consequently, urgent data delivery delay and the failure rate can be minimized; the urgent data packets can be delivered directly from the cluster heads that gathered them from their members to the sink node without a routing process. Hence, considering the zone priority in the path planning of the mobile sink can improve the latency and/or throughput of a high-priority data flow. Moreover, it can improve network lifetime by saving the consumed energy during the routing process.

Another challenging issue is that environmental conditions also influence the WSN performance since it is commonly installed in hostile environments, exposing sensor nodes and sink nodes to failure. Thus, the network seldom works. For instance, sensor nodes and sink nodes are more likely to fail at high temperatures and might be entirely burnt out. Moreover, their short-circuit probability would be raised at extremely high humidity [13,14]. Thus, reducing network performance consequences such as temperature and humidity is important. Therefore, the sink node should be prevented from moving through dangerous areas (for example, areas located in a harsh environment). If the mobile sink crosses a risky zone, such as a fire zone, data transmission in the whole network will be cut off due to burned sink node. Consequently, environmental awareness should be considered when designing the mobile sink’s path planning algorithm.

Urgent real-time data must be delivered to the mobile sink in real-time (deadline). In this scenario, timely data transmission guarantees that the appropriate actions are made, whereas late data delivery affects the action’s efficacy. Therefore, this issue has to be considered in constructing the path planning of the mobile sink [14,15].

Suppose urgent messages such as sudden emergency events occur in sub-areas where the mobile sink has not yet been visited. In that case, such urgent messages should be delivered immediately to the mobile sink through an efficient routing technique. Since the WSN’s functioning relies heavily on the battery life of its sensor nodes, developing a routing algorithm that balances energy usage and energy awareness is a big challenge. In addition, as described above, it is important to reduce the effects of environmental parameters (e.g., humidity and temperature) on network efficiency. Therefore, the urgent data packets should be transmitted over paths that would cross a dangerous zone, such as a fire zone, which would inevitably interrupt data delivery once the relay nodes in that zone are burned. Thus, environmental awareness is a crucial factor that needs to be considered when designing a routing algorithm in WSNs. Moreover, since urgent data packets must be delivered reliably and on time, the routing algorithm should prioritize reliable transmission while being suitable for real-time applications [14,15].

For resolving the aforementioned problems, nature-inspired algorithms were developed. Swarm intelligence (SI) is one of such algorithms being explored to deliver effective optimization solutions for a wide variety of WSN situations because of their flexibility and adaptability in solving many complex problems. Almost all swarm intelligence optimization approaches are inspired by ant colonies, bees, and animal swarms. The ant colony optimization (ACO) approach resembles social ant colonies. In particular, ants use pheromones as a chemical messenger, and the pheromone concentration is also used to indicate solution quality in ACO to find the ideal solution. Therefore, the problem’s solution is usually related to pheromone concentration [9,16,17]. The work in this paper consists of three phases. The first phase is proposing an efficient means of mobile sink path planning as many researchers based their research on problems related to the path planning of the mobile sink. Most of them also tried to determine the shortest path with the least cost and time [18,19,20,21,22,23,24] without considering the environmental effect, which may be affected by path changes. Path planning under difficult situations is uncertain. Therefore, this paper uses environmental awareness to reduce mobile sink’s environmental effect. Secondly, the consequence of the excessive routing of urgent data would negatively influence the latency and/or throughput of a high-priority data flow. Hence, the zone priority is considered when deciding on a new mobile sink location. In addition, delivery delay and distance are also considered. In the second phase, a cluster head is chosen. The proposed clustering strategy chooses cluster heads from normal sensor nodes based on energy efficiency and network reliability to improve network lifetime and throughput. It considers sensor node residual energy, wireless link quality, and intra-cluster distance.

The third step is the routing algorithm. Up to our knowledge, numerous research publications have examined WSN routing problems. Most suggested solutions aimed at increasing data transmission reliability and energy efficiency [9,25,26]. Such studies do not address the environmental effect in routing protocol design. Therefore, they cannot adapt quickly to environmental disturbances like rainstorms and wildfires. To the best of our knowledge up to the time of writing this paper, only two routing protocols [13,14] considered energy and environment awareness when seeking to increase routing reliability and energy efficiency under hostile situations. The problem with these algorithms is that they cannot be used in real time. So, this paper suggests a routing algorithm which tries to reduce the adverse environmental effects on data delivery and is also suitable for real-time applications. It also looks at the link quality so as not to send data packets down paths that are not reliable. Moreover, a novel function interrelates residual energy, and sensor node load is advocated to balance energy utilization.

The path planning, cluster head selection, and routing problems are formulated in the form of 0/1 integer programming for other researchers to understand and reuse. Then, swarm intelligence is introduced as a heuristic solution for the three problems.

The paper outline is as follows: work relevant to this topic is included in Section 2; Section 3 describes the problem; Section 4 presents the problem formulation; Section 5 explains the proposed algorithms; Section 6 covers the results; and the paper’s conclusions are presented at Section 7.

## 2. Related Work

Researchers have proposed numerous ways to prolong the network lifetime. Mobile sinks are one of the most effective solutions for reducing WSN energy usage and improving network lifetime. Due to its relevance in real-world applications, academics and researchers are interested in mobile sink path planning. WSNs are the foundation of IoT applications; mobile sink path planning is considered one of the key factors underpinning their efficacy.

Recent research divides the sink mobility problem into optimum mobile sink paths with and without clustering. Since the research most related to our proposal is the optimal mobile sink path planning with clustering, this section starts with the description of the algorithms that are mostly related to the proposed solution presented in [18,19,20,21,22,23,24]. Then, it discusses their differences from our proposal as well. Finally, as the research presented in [18,19,20,21,22,23,24] does not address the cluster-based routing problem for inter-cluster communication, which is one of this paper’s main objectives, this section discusses the proposed solution compared to the related research presented in [27,28].

An energy-efficient route planning strategy for wireless sensor networks (WSNs) with several mobile sinks is proposed by the authors in [18]. This method is based on a stable election algorithm (SEA) and clustering algorithms for homogeneous and heterogeneous sensor networks. This work aimed to increase network life and connection. The stable election algorithm reduces message flow between sensor nodes and prevents redundant cluster head rotation. Cluster initiation, head rotation, and data collecting make up the SEA. After sensor node deployment, cluster initiation starts. The sensor node with the most neighbors in a homogeneous network is the initial cluster head. The initial cluster head depends on surrounding nodes and node energy in a heterogeneous network. When residual energy drops below a threshold, cluster heads rotate. The node with the greatest rotation index will be the cluster leader. The sink node receives data from nearby cluster heads. Mobile sink path planning examined sojourn locations using the minimum weighted vertex cover problem (MWVCP). The optimal sink path should visit all sojourn spots; however, an MOEA is used to decrease cost, distance, and time.

The authors of [19] present an evolutionary game-based mobile sink trajectory design algorithm (EGTDA). The model of the evolutionary game considered each cluster’s average residual and inter-cluster energy consumption. The mobile sink travels to a cluster with the highest residual energy and the shortest distance to surrounding clusters. This model proposes a clustering algorithm that compares each node to its neighbors to find the cluster head. When two nodes have the same amount of remaining energy, a smaller node ID is selected. This is done to ensure that each cluster has only one cluster head.

KH-TSP optimizes mobile sink paths in large-scale wireless sensor networks [20]. Iterative filtering is used to aggregate the collected data into cluster heads. K-mean clustering is utilized to group randomly placed sensor nodes. It utilizes residual energy and node proximity to construct second-level clusters. TSP used these clusters to build the optimal path sequence. The sequences number is merged to produce a population for the krill optimization method, where the average delay in each sequence is evaluated. The population was designed to reduce delay. The KH-TSP facilitates large-scale WSN data collection, conserves energy, and extends the network’s lifetime.

The clustering-based movable sink route approach was suggested by the authors of [21]. To minimize the sink distance between sensor nodes, they used fuzzy logic. They divided the field into 16 equal zones and made an attempt to calculate the sensor residual energy that should be taken into account. Fuzzy logic and mobile sink nodes increased network longevity, particularly in large-scale networks.

For efficient data collection, the authors of [22] investigated clustering algorithms using mobile sink nodes. They use a modified LEACH approach as the foundation for their clustering. ACO also determines the most direct route for a mobile sink to go to cluster heads and then return to its starting location. Reduced data loss and increased network longevity were the goals.

A clustered data gathering technique for mobile sink nodes is presented by the authors of [23]. In this algorithm, the entire network is split into grids or cells with equal size. To balance the energies of the sensor nodes, the cluster head is chosen depending on the remaining node energy. Moreover, the chosen cluster head should be close to the grid centroid. To maximize network data gathering and establish the shortest path to each mobile sink, an artificial bee colony technique is used.

Reference [24] suggests a WSN routing method based on ACO with mobile sinks (EARP). The WSN multi-sink cluster paradigm is offered by this energy-saving technology. The cluster head, where ACO is used to calculate the optimum route for the mobile sink, is decided by residual energy and distance from sensor nodes. The authors suggested a trustworthy technique for effective energy. However, after examining earlier studies [18,19,20,21,22,23,24], it is clear that they have the following limitations:

- They ignore environmental consequences. Environmental factors may cause sensor and sink nodes to fail, causing the network to malfunction. Neglecting this challenge might make them unable to adapt to environmental changes like wildfires and rainstorms, impairing network performance. Few research publications have explored the external environment’s detrimental influence on data fusion and delivery [13,14]. Unfortunately, up to our knowledge, no research paper has studied the data collection problem of WSNs with mobile sinks under harsh environmental conditions;
- They ignored zone priority. Messages coming from sensor nodes located in high-priority zones should be transmitted immediately to the sink node. Hence, ignoring zone priority could increase urgent data delivery delay and failure rate, which negatively affects the latency and/or throughput of a high-priority data flow;
- They overlook the dependability of data transmission. WSNs depend on reliable data transfer. Ignoring such concerns might increase packet loss, waste energy, and delay packet retransmission. This clearly impacts network efficiency;
- They miss real-time data transmission. Real-time delivery of data is difficult in WSNs. Transmission of crucial real-time data within a predetermined deadline enables prompt action, whereas late delivery reduces the efficacy of the action done. Ignoring this problem might increase missed deadlines and packet drops. Table 1 provides an overall comparison of the algorithms mentioned earlier [18,19,20,21,22,23,24] and the proposed ones.

With regard to the cluster-based routing problem for inter-cluster communication, many academics have investigated this problem, where each cluster head assigns a relay node to forward collected data to the sink node [27,28,29]. In the related protocols [27,28], each cluster head picks its next-hop relay node using a cost function to reduce and balance sensor node energy consumption. Cost function incorporates energy efficiency, residual energy, and distance.

The prior research of similar algorithms demonstrates that they have limitations since they ignore critical issues. The first concern is the environmental effect; disregarding it will instantly cut off WSN’s data delivery. Second, they do not account for lossy links caused by fading and interference; ignoring such a problem might increase data loss, retransmission delay, and energy waste. Finally, they cannot achieve real-time communications; therefore, they cannot send data packets before the deadline. As has been said, timely data supply ensures relevant actions are made, whereas late data delivery reduces the efficacy of such activities. Table 2 provides an overall comparison of the two algorithms mentioned earlier [27,28] and the proposed one.

Motivated by the above discussion, the following points are advanced in this paper:

1. We present a unique data collecting path planning technique that considers environmental effect parameters to reduce mobile sink environmental impact. It achieves this by calculating each node’s environmental effect. Then, it avoids cluster heads in dangerous zones by choosing cluster heads with a higher final environmental impact measure to participate in path planning. Moreover, the zone priority is considered to avoid the excessive routing of urgent data, which would negatively influence the latency and/or throughput of a high-priority data flow. A new zone priority metric function is proposed to integrate the zone priority into the path planning process. The movement cost is thought to reduce the amount of wasted energy during movement, which will make the mobile sink last longer. The movement delay is also considered for real-time urgent data transmission and decreases the chance of buffer overflow of sensor nodes owing to its restricted buffer capacity;
2. Secondly, we propose an energy-efficient clustering technique considering the average intra-cluster distance, residual energy, and reliability of intra-cluster data transmission. In the cluster hierarchy, aggregating the data by each cluster head from its members causes imbalanced energy loss. A novel energy weight function is presented to pick the most energy-efficient node as a cluster head and avoid selecting low-residual-energy nodes. To achieve reliable intra-cluster data transmission, a new link quality metric function is suggested to express the quality of links between each candidate cluster head and its member nodes. We assess the average intra-cluster distance to improve end-to-end latency, as with many previous clustering approaches;
3. Third, we offer a real-time, reliable, energy-efficient, and environment-aware routing approach. The suggested routing strategy minimizes the external environment’s impact on data transmission. Link quality is also considered to prevent data forwarding over unstable paths; a novel function that combines sensor node residual energy and traffic load balances energy usage is proposed. It provides real-time WSN transmission. Only qualified neighbors who can deliver urgent data on time participate in the routing procedure. Moreover, it calculates the relaying delay for each eligible candidate neighbor to reduce route latency. The suggested routing approach uses more realistic parameters than prior systems;
4. Lastly, these problems involve 0/1 integer linear programming. Then, swarm intelligence is proposed for optimization problems.

## 3. Problem Modeling

This section describes the research problems and explains our primary goals. Consider that a field *F(A)* is monitored by a set of sensor nodes for a time horizon *T*. This monitored field is divided into equal-sized zones *A*. A time-varying weight metric is assigned for each zone *i ∈ A*, where *t ∈ T*. This weight metric defines the importance of the observations in each zone over the horizon *T* (surveillance requirements).

In most prior studies, sensor nodes were static and location-aware. Consequently, sink nodes, especially mobile nodes, are utilized to collect the monitored field sensor data. Because of large-scale sensor networks, sensors are organized in the form of clusters. The task of gathering data from each cluster’s members who are located within one hop of it is delegated to a cluster leader. The optimization of sink node mobility comes next. An undirected weighted graph *G (V, E)*, where *V* is the collection of nodes, *E* is the set of edges, *V* is the nodes set, and *E* is the set of edges where *x, y ∈ V*. Vertices represent sensor nodes, whereas edges indicate communication connections between them. An edge only exists between nodes *x* and *y* if they can communicate.

There are some assumptions made concerning the nodes’ characteristics. First, it is assumed that the sensor nodes are equipped with multiple sensor modules (i.e., temperature, humidity), providing sensor nodes with the ability to perceive surrounding environmental information. Secondly, the value of the residual energy can be easily obtained by measuring the batteries’ voltage level. Thirdly, sensor nodes collect the information about their neighbors through broadcasting techniques. Fourthly, it is assumed that the sensor nodes are aware of their geographic locations due to the attached GPS receivers. Finally, the MAC layer PRR [30] measures connection quality.

Let us start with one of the paper’s goals: designing the ideal mobile sink path to improve field coverage. High-priority observations enhance coverage. Moreover, avoiding the risk zone and choosing a safer path for the movable sink to reduce environmental impact. Data packets which did not reach the sink node must also be minimized (packets miss ratio). So, the mobile sink’s path planning algorithm must scan received cluster head locations and choose the optimal position that meets the following restrictions:

(1) Provides minimum movement cost;
(2) Is away from the danger zones to keep mobile sink path safe as much as possible;
(3) Provides the maximum coverage while the observations in the highest priority zones are collected;
(4) Provides the lowest end-to-end latency for real-time data transmission and eliminates sensor node buffer overflow due to limited buffer capacity.

Second, this paper identifies an ideal cluster head that conserves sensor node energy to extend network lifetime. To do this, the cluster head should satisfy some constraints, including:

(1) Provides the shortest average intra-cluster distance;
(2) Provides the highest possible data transfer reliability;
(3) Has the maximum residual energy compared with the other candidates.

Third, this article aims to shorten the routing path’s communication distance to save energy and extend the network’s lifetime. Moreover, energy management is considered while designing the proposed routing protocol. It also examines how to reduce environmental factors’ influence on urgent data packet routing by avoiding hazardous zones and finding the safest paths to the sink. Real-time transmission of urgent data packets is discussed. Additionally, the issue of reliable routing paths is discussed. To attain this aim, the proposed route must fulfill the following criteria:

(1) Minimum communication distance;
(2) Less likely to be cut off as a result of environmental reasons;
(3) Maximum reliability;
(4) Minimum end-to-end delay;
(5) In order to establish a better energy balance, the nodes participating in this path have the highest value resulting from the new proposed energy load function.

## 4. Formulation for Optimal Solution

Due to our optimization expertise, addressing issues optimally makes them simpler to grasp for a better heuristic to be utilized later. In this section, the three problems, namely path planning, clustering, and urgent data routing problems, are mathematically formulated based on integer linear programming (ILP). Although ILP assures an optimal solution and a thorough knowledge of the problem’s s major limitations, large-scale problems are intractable due to time and/or memory requirements. To ensure the efficiency of the given solution, ILP is used to address small-scale problems. To fully understand our solutions’ notations, see Table 3.

### 4.1. Optimal Path Planning Problem

The optimal path planning issue for mobile sinks, which may be categorized as NP-hard optimization problems, is one of the challenges. In order to fulfill the optimal mobile sink route, it must take into account the network delay and cost. A specified set of the best points (locations) in the monitored field must be visited by the best path, and each location must be visited once. As a result, it is clear that there are several limits that must be taken into account.

Let us start with the environmental restriction. WSNs are often deployed in hostile environments, which exposes the sensor nodes and mobile sink to failure and causes the network to malfunction. So, the mobile sink must not cross through danger area. Moreover, the environmental effect on urgent data routing should be lowered as much as possible. To guarantee reliable transmission in harsh environments, such data must not be sent across dangerous places.

To calculate the environmental effect of a single factor f on zone i at time t, apply the following equation:

(1) $$EI_{i}^{f}(t)=\left\{ \begin{array}{l} 1\;\;\;\;\;if\;{ZED}_{L}^{f}\leq {ZED}_{i}^{f}\leq {ZED}_{H}^{f}\\ \frac{ZED_{H}^{f}-ZED_{i}^{f}}{{ZED}_{\max}^{f}-{ZED}_{H}^{f}}+1\;if\;{ZED}_{i}^{f}>{ZED}_{H}^{f}\\ \frac{{ZED}_{i}^{f}-{ZED}_{L}^{f}}{{ZED}_{L}^{f}-{ZED}_{\min}^{f}}+1\;if\;{ZED}_{i}^{f}<{ZED}_{L}^{f} \end{array}\right..$$

**Figure 1:**
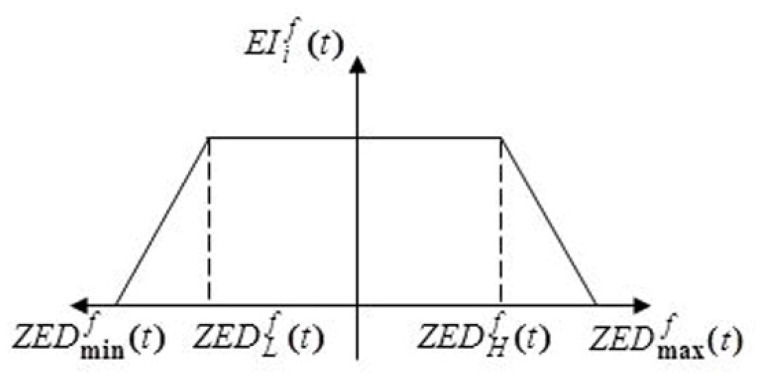
The curve of the single environmental impact metric function.

Inspired by [14], Equation (1) shows the following scenario:

Look at the environmental data value of any zone *i* for such environmental factor *f*, and check the normal operating range $\left(ED_{L}^{f},ED_{H}^{f}\right)$; if it is achieved, it means that the sensor nodes performance in that zone would not be influenced by environmental factor f. Then define the environmental effect metric $EI_{i}^{f}(t)$ to 1. Suppose zone *i’s* environmental data are beyond the typical range. In that case, environmental factor *f* will detrimentally affect the sensor nodes in that zone, causing them to deteriorate and increasing the risk of malfunctions. Therefore, $EI_{i}^{f}(t)$ is decreased. The trend of the given function in Equation (1) which represents the environment single factor impact metric is shown in Figure 1.

Many environmental factors influence the performance of WSNs. So, the environmental impact metric should be determined for multiple environmental factors as follows [13]:

(2) $$MEI_{i}(t)=\min\left\{EI_{i}^{f_{1}}(t),EI_{i}^{f_{2}}(t)\right\}$$

where are the single environmental impact metrics of zone *i* at time *t* with respect to the environmental factors *f_1_* and *f_2_*, respectively. Indeed, suppose the sensor nodes performance in a certain zone would be severely affected by one of multiple environmental factors. In that case, the sink node should be prevented from crossing that zone. Moreover, its sensor nodes should be prevented from being selected as a next-hop through the routing process of the urgent messages. Therefore, among the environmental metrics values of different factors, the multiple environmental impact metric is represented by the minimum value [13].

On the other hand, if such a wildfire occurs in a particular zone, it is expected to spread rapidly to neighboring zones; neighboring zones are still considered hazard zones even though this incident did not spread to them. To keep the mobile sink path and routing paths of urgent data away from environmental risks as early as possible, it is important to link each zone’s environmental effect metric with its neighbors. A zone’s environmental impact on zone *j* is defined as follows: [13]:

(3) $$NEI_{ij}(t)=K_{ij}MEI_{j}(t).$$

The attenuation coefficient *K_ij_* was calculated as follows:

(4) $$K_{ij}=1+\frac{ED_{ij}-ED_{ij}{}_{\min}}{ED_{ij}{}_{\max}-ED_{ij}{}_{\min}}$$

where $ED_{ij}{}_{\min}$ and $ED_{ij}{}_{\max}$ represent the minimum and maximum distance between the center of zone *i* and *j*. For a square/circular zone with side length/diameter *a*, $ED_{ij}{}_{\min}$ and $ED_{ij}{}_{\max}$ could be calculated as follows:

(5) $${ED}_{ij}{}_{\min}=a$$

(6) $${ED}_{ij}{}_{\max}=2\sqrt{2}a.$$

For rectangular shaped zone with side length *L* and side width *w*, $ED_{ij}{}_{\min}$ and $ED_{ij}{}_{\max}$ could be calculated as follows:

(7) $${ED}_{ij}{}_{\min}=L$$

(8) $${ED}_{ij}{}_{\max}=2\sqrt{L^{2}+w^{2}}.$$

Equations (3) and (4) show that the attenuation coefficient is proportional to zone *i* and *j* distance. This is because each zone is more sensitive to environmental threats from its neighbors when they are closer. As numerous bordering zones surround each zone, each has its own neighboring environmental effect metric. The smallest value among all adjacent metrics is selected as the final neighboring environmental impact as follows:

(9) $$NEI_{i}(t)=\min\left\{NEI_{ij}(t)|{}_{j\in NZ_{i}}\right\}.$$

Finally, the final environmental impact of zone *i* is jointly calculated by the multiple environmental impacts $MEI_{i}(t)$ and the neighboring environmental impact $NEI_{i}(t)$ as follows:

(10) $$FEI_{i}(t)=\left\{ \begin{array}{l} MEI_{i}(t)\;\;\;\;\;\;if\;NEI_{i}(t)=1\\ \frac{MEI_{i}(t)+NEI_{i}(t)}{2+\exp(-K_{ij})}\;\;\;\;otherwise \end{array}\right..$$

According to Equation (10), the proposed final environmental effect metric is based on the insignificant nearby environmental impact during normal operation periods. If a neighbor’s environmental effect is one, it is appropriate to ignore it. As a zone’s neighbor’s attenuation coefficient drops, so should its final environmental metric. This means the closest neighborhood zone will have a larger environmental impact. So, the final environmental metric should be proportional to the attenuation coefficient. Finally, the exponential function is used where a small value of the attenuation coefficient results in a reasonably large decrease in the proposed metric result.

We must assess the impact of the environmental characteristics on each cluster head since the mobile sink’s path planning algorithm must seek cluster head locations to avoid risky regions. According to Equation (11), the following is the ultimate environmental effect measure for a cluster head *x* in zone *i*:

(11) $$EIM_{x}^{i}(t)=FEI_{i}(t).$$

For the path planning algorithm to circumvent the cluster heads in the danger zones, the cluster heads in the candidate cluster head set $CH^{t}$ that have a final environmental impact metric larger than the threshold value are added to the final candidate cluster head set $FCH^{t}$.

The path planning method avoids cluster heads in the candidate cluster head set $CH^{t}$ in hazardous zones by adding cluster heads with high final environmental effect metrics to the final candidate cluster headset $FCH^{t}$; formally, as in Equation (12):

(12) $$FCH^{t}=\left\{x|x\in CH^{t},EIM_{x}^{i}\left(t\right)>EI_{th}\right\}.$$

**Figure 2:**
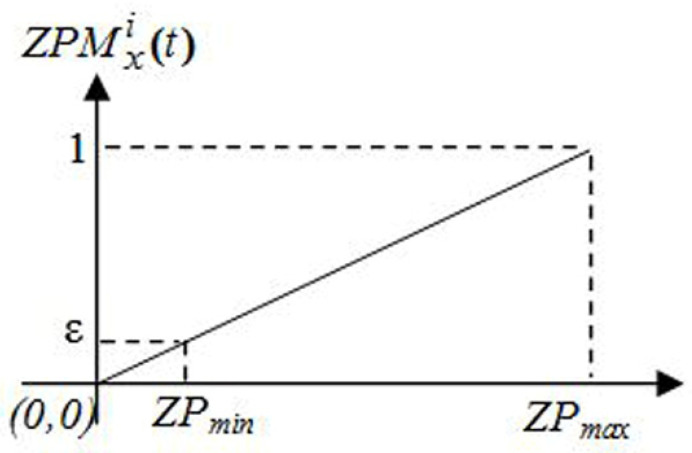
Function curve for zone priority.

A heterogeneous environment is often used for deploying the WSN. As a result, the environment is divided into discrete regions known as zones, and these regions are then classified by assigning each zone a value known as zone priority based on its given priority (important) [12]. Data that is urgent is sent via sensor nodes located in high-priority areas. Urgent data packets should be sent to the sink right away since they have a high priority. The network topology is clustering-based, and the sink node rotates regularly among cluster heads. Imagine that a certain cluster head in a high-priority zone collects urgent data packets from its members when the sink is either visiting it for the first time or has previously done so. In such scenario, the routing method should be used to quickly convey this urgent data to the sink. Since the mobile sink will go first to high-priority cluster heads, zone priority is an important factor to take into account while determining the mobile sink’s path. As a result, urgent data may be transferred directly from cluster heads to sink nodes without the need for routing. Therefore, excessive route avoidance may reduce network lifespan and/or increase latency and/or throughput of critical data. In order to find the optimal data collection path for the mobile sink utilizing a new function called the zone priority metric function, we incorporate zone priority as one of the key metrics. Equation (13) defines the zone priority measure for a cluster head *x* in zone *i*:

(13) $$ZPM_{x}^{i}(t)=\frac{\left(1-\varepsilon \right)}{ZP_{\max}-ZP_{\min}}ZP_{i}(t)$$

where, *ZP_min_* and *ZP_max_* are the minimum and maximum values assigned for zone priority. The zone priority function gives a high-priority cluster head a better chance of becoming the movable sink’s new location. To analyze the validity of the selected trend for the zone priority function curve in Figure 2, we consider that, to find the best path for a mobile sink, MS, it is necessary to move among three cluster heads, as an example. Assume zone priorities of 10, 5, and 0 for the three cluster heads, respectively. The minimum and maximum zone priority values are 1 and 10, and the value of *ε* is set to 0.1. The sink node should shift to the highest priority cluster head. Applying the zone priority function yields the values 1, 0.5, and 0. Cluster heads with the greatest priority have the highest value, while those with no importance have zero. This suggested function moves the mobile sink to the highest priority cluster head.

Urgent, real-time data must be sent to the mobile sink within predefined deadlines; performance is assessed by how many packets are received before the deadline. Therefore, Urgent messages must be transmitted with the proper latency, reducing packet loss. Equation (14) defines the required message delivery latency for cluster head *x* [31]:

(14) $$Dd_{x}=\frac{Deadline}{Hc_{\left(x,MS\right)}(t)}.$$

According to Equation (14), latency is inversely proportional to sink node distance. As the sink’s distance grows, the required delay lowers. Therefore, critical messages will not arrive in time. Consequently, the increase in lost packets affects real-time performance. We estimate distance using hops. Path planning must minimize the distance from the sink node to cluster heads with urgent messages to boost real-time performance. The cluster head with the shortest distance should be the new mobile sink location, according to this article’s analysis of the average distance between cluster heads carrying urgent messages. The average distance measure is provided by Equation (15) as follows for a certain cluster head *x*:

(15) $$Ad_{x}=\frac{\sum_{l\in CHU^{t}}ED_{xl}}{\left|CHU^{t}\right|}$$

where |.| denotes the size of a set.

The created method sought to concurrently delay the mobile sink path and decrease movement cost (measured in terms of movement energy).

The sum of all edge costs and delay along the path in graph *G* represents the overall movement cost and delay of the valid path *p* for the mobile sink *MS*:

(16) $$Total\;\mathrm{movement}\;\cos t\;(P,G)=\sum_{t\in T}\sum_{u,x\in FCH^{t}}\xi_{ux}^{t}C_{ux}\left(t\right)$$

(17) $$Total\;\mathrm{movement}\;\mathrm{delay}\;(P,G)=\sum_{t\in T}\sum_{u,x\in FCH^{t}}\xi_{ux}^{t}D_{ux}\left(t\right).$$

Based on these computations, Equations (18) and (19) objective functions minimize cost and delay, respectively:

(18) $$Z_{IP_{1}}=\min\sum_{t\in T}\sum_{u,x\in FCH^{t}}\xi_{ux}^{t}C_{ux}^{t}$$

(19) $$Z_{IP_{2}}=\min\sum_{t\in T}\sum_{u,x\in FCH^{t}}\xi_{ux}^{t}D_{ux}^{t}$$

subject to:

(20) $$\sum_{x\in CH^{t}}r_{x}EIM_{x}^{t}\left(t\right)>EI_{th}\;\;\;\;CH^{t}\in V\;$$

(21) $$\sum_{x\in CH^{t}}r_{x}\geq 1\;\;\;\;CH^{t}\in V$$

(22) $$\sum_{x\in FCH^{t}}X_{ux}^{t}\leq 1\;\;\;\;\forall t\in T,u\in FCH^{t},FCH^{t}\in CH^{t}{,CH}^{t}\in V\;$$

(23) $$\sum_{n\in FCH^{t}-\{x\}}E_{n}\left({EIM}_{x}^{i}\left(t\right)-EIM_{n}^{j}\left(t\right)\right)<0\;\;\;\;\;\forall x\in FCH^{t},i,j\in Z,t\in T,,FCH^{t}\in CH^{t}{,CH}^{t}\in V$$

(24) $$2-\sum_{n\in FCH^{t}-\{x\}}E_{n}=I_{x}+1\;\;\;\;\forall x\in FCH^{t},FCH^{t}\in CH^{t}{,CH}^{t}\in V$$

(25) $$\sum_{n\in FCH^{t}-\{x\}}E_{n}\leq 1\;\;\;\;\forall x\in FCH^{t}{,FCH}^{t}\in {CH}^{t},CH^{t}\in V\;$$

(26) $$\sum_{x\in FCH^{t}}I_{x}\leq 1\;\;\;\;{\;FCH}^{t}\in CH^{t},CH^{t}\in V\;$$

(27) $$\sum_{\upsilon \in FCH^{t}-\{x\}}g_{\upsilon }(ZPM_{x}^{i}\left(t\right)-ZPM_{\upsilon }^{j}\left(t\right))<0\;\;\;\;\forall x\in FCH^{t}{,FCH}^{t}\in {CH}^{t},CH^{t}\in V,t\in T$$

(28) $$2-\sum_{\upsilon \in FCH^{t}-\{x\}}g_{\upsilon }=P_{x}+1\;\;\;\;\forall x\in FCH^{t}{,FCH}^{t}\in {CH}^{t},CH^{t}\in V$$

(29) $$\sum_{\upsilon \in FCH^{t}-\{x\}}g_{\upsilon }\leq 1\;\;\;\;\forall x\in FCH^{t}{,FCH}^{t}\in {CH}^{t},CH^{t}\in V\;$$

(30) $$\sum_{x\in FCH^{t}}P_{x}\leq 1\;\;\;\;{\;FCH}^{t}\in {CH}^{t},CH^{t}\in V\;$$

(31) $$\sum_{k\in FCH^{t}-\{x\}}h_{k}\left(Ad_{k}-Ad_{x}\right)<0\;\;\;\;\forall x\in FCH^{t}{,FCH}^{t}\in {CH}^{t},CH^{t}\in V$$

(32) $$2-\sum_{k\in FCH^{t}-\{x\}}h_{k}=m_{x}+1\;\;\;\;\forall x\in FCH^{t}{,FCH}^{t}\in {CH}^{t},CH^{t}\in V$$

(33) $$\sum_{k\in FCH^{t}-\{x\}}h_{k}\leq 1\;\;\;\;\forall x\in FCH^{t}{,FCH}^{t}\in {CH}^{t},CH^{t}\in V\;$$

(34) $$\sum_{x\in FCH^{t}}m_{x}\leq 1\;\;\;\;{\;FCH}^{t}\in {CH}^{t},CH^{t}\in V\;$$

(35) $$\sum_{x\in FCH^{t}}I_{x}P_{x}m_{x}\leq X_{ux}^{t}\;\;\;\;\forall t\in T,u\in FCH^{t}{,FCH}^{t}\in {CH}^{t},CH^{t}\in V$$

(36) $$\sum_{u,x\in FCH^{t}}\xi_{ux}^{t}\geq 1\;\;\;\;\forall t\in T,{FCH}^{t}\in {CH}^{t},CH^{t}\in V$$

(37) $$\begin{array}{l} \left\{\xi_{ux}^{t},X_{ux}^{t},r_{x},I_{x},P_{x},m_{x},E_{n},g_{\upsilon },h_{k}\right\}=0\;or\;1\;\\ \forall t\in T,u,x\in FCH^{t},n,\upsilon ,k\in FCH^{t}-\{x\},FCH^{t}\in CH^{t},CH^{t}\in V \end{array}$$

Constraints in (20) and (21) ensure that the final candidate cluster head set $FCH^{t}$ contains only the cluster heads with a final environmental impact metric larger than the threshold value; it is often less members than $CH^{t}$. If no cluster heads in the candidate set $CH^{t}$ meet this condition, the $FCH^{t}$ will not have a member. Therefore, these constraints are also used to ensure that $FCH^{t}$ has members; otherwise, the network fails.

The constraint in (22) is intended to prevent any cycle. Cluster head *x* for the mobile sink costs 1 for each time period selected for the following location. The mobile sink is moved away from danger by the constraints in (23) though (26), which guarantee that cluster head *x* is the mobile sink’s next position. The movable sink is relocated to the cluster heads in the high-priority zones first due to constraints (27) through (30). By deciding on cluster head *x* as the next location for the mobile sink, the maximum zone priority measure is met. The constraints in (31) to (34) make sure that choosing cluster head *x* as the mobile sink’s next location meets a minimum average distance metric so that critical data packets must arrive to the sink by a certain time.

The constraint in (35) ensures that the decision variable $X_{ux}^{t}$ is set to 1 when the cluster head *x* has the maximum value for both metrics ${EIM}_{x}^{i}\left(t\right)$ and $ZPM_{x}^{i}\left(t\right)$ and the minimum value of $Ad_{x}^{i}$ compared to other cluster heads. The constraint in (36) is an iterative constraint where all $\sum_{u,x\in CH^{}}\xi_{ux}^{t}\geq 1$ must be greater than or equal to 1. The constraint in (37) ensures that the decision variables $\xi_{ux}^{t},X_{ux}^{t},I_{x},P_{x},m_{x},E_{n},g_{\upsilon },h_{k}$ are 0 or 1.

### 4.2. Optimal Cluster Head Selection Problem

The second problem in this paper is how to select the optimal cluster head with the objective of minimizing the intra-cluster distance. A set of constraints is defined for such optimization problems, including energy and reliability constraints.

For each node *x*, the nodes in its candidate neighbor set *NEB_x_* that are not covered by any cluster head are added to the final candidate member set $NM_{x}$; formally, as in Equation (38):

(38) $$NM_{x}=\left\{y|y\in NEB_{x},\Gamma_{y}=1\right\}$$

where

(39) $$\Gamma_{y}=\{\begin{array}{cc} 1 & \;\mathrm{if}\;\mathrm{node}\;y\;\mathrm{is}\;\mathrm{not}\;\mathrm{covered}\;\mathrm{by}\;\mathrm{any}\;\mathrm{cluster}\;\mathrm{head}\\ 0 & \mathrm{otherwise} \end{array}.$$

As WSNs rely on reliable data transmission, dependable wireless networks are needed to transfer data packets from member nodes to cluster heads. However, in a WSN, the packet losses are quite inevitable since it is being deployed in harsh environmental conditions. As a result, it needs more time and energy to retransmit lost packets, which degrades timely data delivery and network lifetime [6,7,8,9]. The proposed clustering technique incorporates link quality into the cluster heads selection decision to overcome this problem and improve network throughput. In addition, the retransmission reduction decreases energy consumption, which enhances network lifetime. Equation (40) presents the proposed new link quality metric of candidate cluster head *x* at time *t*:

(40) $$QM_{x}=\sum_{l\in NM_{x}}\left(1-PRR_{xl}\right).$$

According to Equation (40), the proposed quality metric function is designed to express the quality of links between the candidate cluster head *x* and its neighbor nodes *NEB_x_*. Moreover, this is designed because the maximum link quality value is 1. Hence, through the quality metric function, the closer the link quality value is to 1, the lower the resulting value for the proposed function, leading to choosing the cluster head that can provide the more reliable links for data transmission from its members.

Each cluster’s head receives, aggregates, and sends data to the sink. Therefore, cluster heads lose energy quicker than sensors. To balance energy consumption across all nodes and extend network lifetime, the clustering must avoid low-residual-energy nodes. Therefore, each cluster head’s energy weight should be an exponential function of node residual energy, Equation (41):

(41) $$EM_{x}\left(t\right)=\exp\left(\frac{RE_{x}\left(t\right)}{IE_{x}}-1\right).$$

As indicated in Equation (41), the energy metric of each candidate cluster head is constructed because the exponential function is a sort of function where tiny changes in variables may produce big changes in function values [32]. This kind of function is used to build an energy weight function. Therefore, when sensor nodes’ residual energy fluctuates with a minor value, it will result in a considerable variation in the proposed function’s output. This should result in the best energy cluster head being chosen. Reduced intra-cluster distance is the goal. It is described as the sum of the average distances between each member node and all selected cluster heads throughout all time periods as given in Equation (42):

(42) $$the\;\mathrm{over}\;\mathrm{all}\;\mathrm{average}\;\mathrm{intra}-\mathrm{cluster}\;\mathrm{distance}=\sum_{t\in T}\sum_{x\in V}\psi_{x}\frac{\sum_{y\in NM_{x}}ED_{xy}}{\left|NM_{x}\right|}.$$

Based on this computation, the problem is formulated as follows:

(43) $$Z_{\mathrm{IP}}=\min\sum_{t\in T}\sum_{x\in V}\psi_{x}\frac{\sum_{y\in NM_{x}}ED_{xy}}{\left|NM_{x}\right|}$$

subject to:

(44) $$\sum_{x\in V}\delta_{x}^{t}\;\leq 1\;\;\;\;\;\;\;\;\;\;\;\forall t\in T$$

(45) $$\sum_{f\in V-\{x\}}\varepsilon_{f}\left(QM_{f}-QM_{x}\right)<0\;\;\;\;\forall x\in V$$

(46) $$2-\sum_{f\in V-\{x\}}\varepsilon_{f}=q_{x}+1\;\;\;\;\;\;\forall x\in V$$

(47) $$\sum_{f\in V-\{x\}}\varepsilon_{f}\leq 1\;\;\;\;\;\;\;\;\;\;\forall x\in \in V\;$$

(48) $$\sum_{x\in V}q_{x}\leq 1$$

(49) $$\sum_{b\in V-\{x\}}\mu_{b}\left(EM_{x}\left(t\right)-EM_{b}\left(t\right)\right)<0\;\;\;\;\forall x\in V,\forall t\in T$$

(50) $$2-\sum_{b\in V-\{x\}}\mu_{b}=R_{x}+1\;\;\;\;\;\;\forall x\in V$$

(51) $$\sum_{b\in V-\{x\}}\mu_{b}\leq 1\;\;\;\;\;\;\;\;\;\;\forall x\in \in V\;$$

(52) $$\sum_{x\in V}R_{x}\leq 1$$

(53) $$\sum_{x\in V}q_{x}R_{x}\leq \delta_{x}^{t}\;\;\;\;\;\;\;\forall t\in T$$

(54) $$\sum_{x\in V}\psi_{x}\geq 1$$

(55) $$\left\{\psi_{x},\varepsilon_{f},q_{x},\mu_{b},R_{x}\right\}=0\;or\;1\;\;\;\;\;\forall x,f,b\in V$$

The constraint in (44) ensures that any node *x* is covering at most one cluster at each time interval. Constraints in (45) through (48) guarantee that the selection of a cluster head *x* satisfies the minimum quality metric condition where the data packets from the cluster members are collected and aggregated on a reliable link. The constraints in (49) through (52) manage sensor node energy consumption to balance and preserve residual energy, extending the network’s lifetime. The selected cluster head *x* must have the maximum value of energy metric compared with other candidate nodes. The constraint in (53) ensures that the decision variable $\delta_{x}^{t}$ is enforced to 1 when node *x* has the maximum value of the metric ${EM}_{x}\left(t\right)$ and the minimum value $QM_{x}$ compared with other nodes. The constraint in (54) is a redundancy constraint where all $\sum_{x\in V}\psi_{x}\geq 1$ must be greater than or equal to 1. The constraint in (55) ensures that the decision variables $\left\{\psi_{x},\varepsilon_{f},q_{x},\mu_{b},R_{x}\right\}$ are equal to 0 or 1.

### 4.3. Optimal Routing Problem

The developed routing algorithm aimed at minimizing packet loss ratio and end-to-end delay of the routing path simultaneously. In case urgent data messages occur in some areas that the mobile sink has already visited or has not visited yet, it should be delivered promptly, and thus the routing procedure is invoked. Hence, this paper’s third problem is finding the optimal route for urgent data transmission. A set of constraints is defined for such optimization problems, including energy consumption balance, environmental impact minimization, real-time data delivery, and reliability of data transmission.

It is a known fact that in WSNs the communication energy is related to the transmission distance. The quickest way considerably decreases energy use. However, the shortest path approach did not prolong the network’s lifetime. The network’s energy usage must be balanced for energy-efficient routing.

Relying entirely on sensor nodes’ residual energy is not the ideal way to establish network energy balance [14]. The routing protocol must refrain from using low-energy, high-traffic nodes as next-hops in order to achieve a better energy balance. By using the recommended new function, the energy load function, it is feasible to include the remaining energy and traffic load of sensor nodes and let them have a significant impact on choosing the next hop [14].

The total number of messages sent by each cluster head should match that of the node. This should contain the number of messages from each cluster head member and other cluster heads to relay. Equation (56) shows cluster head *y’s* traffic load at time *t*.

(56) $$NL_{y}(t)=\{\begin{array}{cc} TM_{y}\left(t\right) & if{\;ED}_{\left(y,MS\right)}\left(t\right)\leq {ED}_{\left(x,MS\right)}\left(t\right)\\ 0 & otherwise \end{array}$$

(57) ECLy(t)=1exp(IEy−(REy(t)−(NLx(t)*HExy))IEy)

Equation (57) suggested that the new energy consumption load function expresses cluster head *y’s* energy use after sending all messages. Any tiny change in the exponential function input causes a huge output change. By exponential function, a slight change in nodes’ energy leads to the selection of the most energy-efficient relay node [32].

The cluster head that can transmit data packets quicker than other candidates and fulfil the requisite latency should be the next hop. Therefore, it is appropriate to employ relaying delay to support routing decisions. The relaying latency for each candidate node is determined by Equation (58):

(58) $$Rd_{y}(t)=delay_{xy}.$$

For every cluster head *x*, the cluster heads in its candidate neighbor set $CCHN_{x}$ that have relaying delays less than the desired delay are added to the final candidate neighbor set $FCHN_{x}$; formally, as in Equation (59):

(59) $$FCHN_{x}=\left\{y|y\in CCHN_{x},Rd_{y}<Dd_{x}\right\}.$$

As can be seen in Equations (60) and (61), the overall end-to-end delay and packet loss ratio of the routing paths from all cluster heads that have urgent messages $CHU^{t}$ to the mobile sink *MS* in a graph *G* are defined as the summation of all edge contributions along the routing path:

(60) $$Total\;\mathrm{packet}\;\mathrm{loss}\;\mathrm{ratio}\;(G,FCH^{t})=\sum_{t\in T}\sum_{s\in CHU^{t}}\sum_{u\in UM_{s}^{t}}\sum_{x,y\in FCH^{t}}d_{xy}^{su}PLR_{xy}$$

(61) $$Total\;\mathrm{end}-\mathrm{to}-\mathrm{end}\;\mathrm{delay}\;(G,FCH^{t})=\sum_{t\in T}\sum_{s\in CHU^{t}}\sum_{u\in UM_{s}^{t}}\sum_{x,y\in FCH^{t}}d_{xy}^{su}D_{xy}.$$

Based on these calculations, the problem is formulated as follows:

(62) $$Z_{IP_{1}}=\min\sum_{t\in T}\sum_{s\in CHU^{t}}\sum_{u\in UM_{s}^{t}}\sum_{x,y\in FCH^{t}}d_{xy}^{su}PLR_{xy}$$

(63) $$Z_{IP_{2}}=\min\sum_{t\in T}\sum_{s\in CHU^{t}}\sum_{u\in UM_{s}^{t}}\sum_{x,y\in FCH^{t}}d_{xy}^{su}D_{xy}$$

subject to:

(64) $$\sum_{y\in CCHN_{x}}c_{y}Rd_{y}<Dd_{x}\;\;x,CCHN_{x}\in FCH^{t}{,FCH}^{t}\in {CH}^{t}{,CH}^{t}\in V\;$$

(65) $$\sum_{y\in CCHN_{x}}c_{y}\geq 1\;\;\;\;x,CCHN_{x}\in FCH^{t}{,FCH}^{t}\in {CH}^{t}{,CH}^{t}\in V\;$$

(66) $$\sum_{y\in FCHN_{x}}o_{y}\left(\sum_{p\in P_{y}^{t}}\alpha_{y}^{p}\right)>{0\;\;FCHN}_{x}\in {FCH}^{t}\in {CH}^{t}{,CH}^{t}\in V,t\in T$$

(67) $$\sum_{y\in FCHN_{x}}o_{y}\leq 1\;\;\;\;{\;FCHN}_{x}\in {FCH}^{t}\in {CH}^{t}{,CH}^{t}\in V,t\in T\;$$

(68) $$\sum_{x\in FCHN_{x}}\omega_{xy}^{su}\leq 1\;\;\forall s\in CHU^{t},\forall u\in UM_{s}^{t},\forall y\in FCHN_{x},CHU^{t},FCHN_{x}\in {FCH}^{t},t\in T$$

(69) $$\begin{array}{l} \sum_{l\in FCHN_{x}-\{y\}}a_{l}\left(Rd_{l}\left(t\right)-Rd_{y}\left(t\right)\right)<0\;\\ \;\;\forall y\in FCHN_{x},FCHN_{x}\in FCH^{t},FCH^{t}\in CH^{t},CH^{t}\in V,\forall t\in T \end{array}$$

(70) $$\begin{array}{l} 2-\sum_{l\in FCHN_{x}-\{y\}}a_{l}=de_{y}+1\\ \;\;\;\forall y\in FCHN_{x},FCHN_{x}\in FCH^{t},FCH^{t}\in CH^{t},CH^{t}\in V,\forall t\in T \end{array}$$

(71) $$\sum_{l\in FCHN_{x}-\{y\}}a_{l}\leq 1\;\;\forall y\in FCHN_{x},FCHN_{x}\in FCH^{t},FCH^{t}\in CH^{t},CH^{t}\in V,\forall t\in T$$

(72) $$\sum_{y\in FCHN_{x}}de_{y}\leq 1\;\;\;FCHN_{x}\in FCH^{t},FCH^{t}\in CH^{t},CH^{t}\in V,\forall t\in T$$

(73) $$\begin{array}{l} \sum_{\upsilon \in FCHN_{x}-\{y\}}b_{\upsilon }\left(ECL_{y}\left(t\right)-ECL_{\upsilon }\left(t\right)\right)<0\;\\ \;\;\;\forall y\in FCHN_{x},FCHN_{x}\in FCH^{t},FCH^{t}\in CH^{t},CH^{t}\in V,\forall t\in T \end{array}$$

(74) $$\begin{array}{l} 2-\sum_{\upsilon \in FCHN_{x}-\{y\}}b_{\upsilon }=e_{y}+1\\ \;\forall y\in FCHN_{x},FCHN_{x}\in FCH^{t},FCH^{t}\in CH^{t},CH^{t}\in V,\forall t\in T \end{array}$$

(75) $$\sum_{\upsilon \in FCHN_{x}-\{y\}}b_{\upsilon }\leq 1\;\;\forall y\in FCHN_{x},FCHN_{x}\in FCH^{t},FCH^{t}\in CH^{t},CH^{t}\in V,\forall t\in T$$

(76) $$\sum_{y\in FCHN_{x}}e_{y}\leq 1\;\;\;\;FCHN_{x}\in FCH^{t},FCH^{t}\in CH^{t},CH^{t}\in V,\forall t\in T$$

(77) $$\begin{array}{l} \sum_{n\in FCHN_{x}-\{y\}}v_{n}\left(EIM_{y}^{i}\left(t\right)-EIM_{n}^{j}\left(t\right)\right)<0\;\\ \;\;\;\forall y\in FCHN_{x},FCHN_{x}\in FCH^{t},FCH^{t}\in CH^{t},CH^{t}\in V,\forall t\in T \end{array}$$

(78) $$\begin{array}{l} 2-\sum_{n\in FCHN_{x}-\{y\}}v_{n}=N_{y}+1\\ \;\;\forall y\in FCHN_{x},FCHN_{x}\in FCH^{t},FCH^{t}\in CH^{t},CH^{t}\in V,\forall t\in T \end{array}$$

(79) $$\sum_{n\in FCHN_{x}-\{y\}}v_{n}\leq 1\;\;\;\forall y\in FCHN_{x},FCHN_{x}\in FCH^{t},FCH^{t}\in CH^{t},CH^{t}\in V,\forall t\in T$$

(80) $$\sum_{y\in FCHN_{x}}N_{y}\leq 1\;\;FCHN_{x}\in FCH^{t},FCH^{t}\in CH^{t},CH^{t}\in V,\forall t\in T$$

(81) $$\begin{array}{l} \sum_{y\in FCHN_{x}}c_{y}o_{y}e_{y}N_{y}\leq \omega_{xy}^{su}\\ \;\forall s\in CHU^{t},\forall u\in UM_{s}^{t},x,CHU^{t},FCHN_{x}\in FCH^{t},FCH^{t}\in CH^{t},t\in T \end{array}$$

(82) $$\sum_{x,y\in FCH^{t}}d_{xy}^{su}\geq 1\;\;\;\forall s\in S,\forall u\in UM_{s}^{t},FCH^{t}\in CH^{t},CH^{t}\in V,t\in T$$

(83) $$\sum_{p\in P_{y}^{t}}\alpha_{y}^{p}\geq 1\;\;\forall y\in FCHN_{x},FCHN_{x},x\in FCH^{t},FCH^{t}\in CH^{t},CH^{t}\in V,t\in T$$

(84) $$\begin{array}{l} \left\{d_{xy}^{su},\alpha_{y}^{p},\omega_{xy}^{su},c_{y},o_{y},b_{\upsilon },e_{y},v_{n},N_{y}\right\}=0\;or\;1\;\\ \forall s\in CHU^{t},\forall u\in UM_{s}^{t},x,n,\nu ,CHU^{t}\in FCH^{t},FCH^{t}\in CH^{t},t\in T \end{array}$$

Constraints in (64) and (65) are utilized to ensure that the forwarding candidate set $FCHN_{x}$ contains only the cluster heads that have a relay delay less than the desired delay; it often has less members than $CCHN_{x}$. If there is no cluster head in the candidate set $CCHN_{x}$ which satisfies this condition, the $FCHN_{x}$ will not have a member. Therefore, such constraints are used to ensure the presence of members in set $FCHN_{x}$; otherwise, drop control is called. Constraints in (66) and (67) ensure that every cluster head *y* reaches the mobile sink. Any cluster head *y* must be on at least one path to the sink. The constraint in (68) is utilized to avoid cycles; for the same source cluster head *s* and urgent message *u*, the use of any cluster head *y* as a relay node has a cost of 1. Constraints in (69) through (72) ensure that only one cluster head *y* meets the minimum relaying delay criterion while urgent data packets are delivered to the mobile sink on time. The constraints in (73) through (76) ensure energy consumption balance to prolong network lifetime. The only cluster head *y* that has the highest value of $ECL_{y}(t)$ must be selected from the final set of the cluster head neighbors of cluster head *x*. Constraints in (77) through (80) ensure that the cluster head *x* picks only one cluster head *y* from its final cluster head neighbor set that meets the maximum final environmental impact metric condition so that data packets are sent to the mobile sink in safe paths away from hazards.

The constraint in (81) is utilized to ensure that the decision variable $\omega_{xy}^{su}$ is enforced to 1 when: (1) the cluster head *y* reaches mobile sink, (ii) it has the minimum value of $Rd_{y}$ compared with other cluster head neighbors, and (iii) it has the maximum value of $EIM_{y}^{i}(t)$ and $ECL_{y}(t)$ compared with other cluster head neighbors. Constraints in (82) through (83) are redundancy constraints where all $\sum_{x,y\in CH^{t}}d_{xy}^{su}$ and $\sum_{p\in P_{y}^{t}}\alpha_{y}^{p}$ must be greater than or equal to 1. The constraint in (84) ensures the decision variables $\left\{d_{xy}^{su},\alpha_{y}^{p},\omega_{xy}^{su},c_{y},o_{y},b_{\upsilon },e_{y},v_{n},N_{y}\right\}$ are equal to 0 or 1.

## 5. Swarm Based Solution

This section presents greedy algorithms as the optimum methods discussed previously are not applicable for large-scale WSNs. The suggested algorithms are for small- and large-scale real-time WSNs.

### 5.1. Path Planning Problem

**Figure 3:**
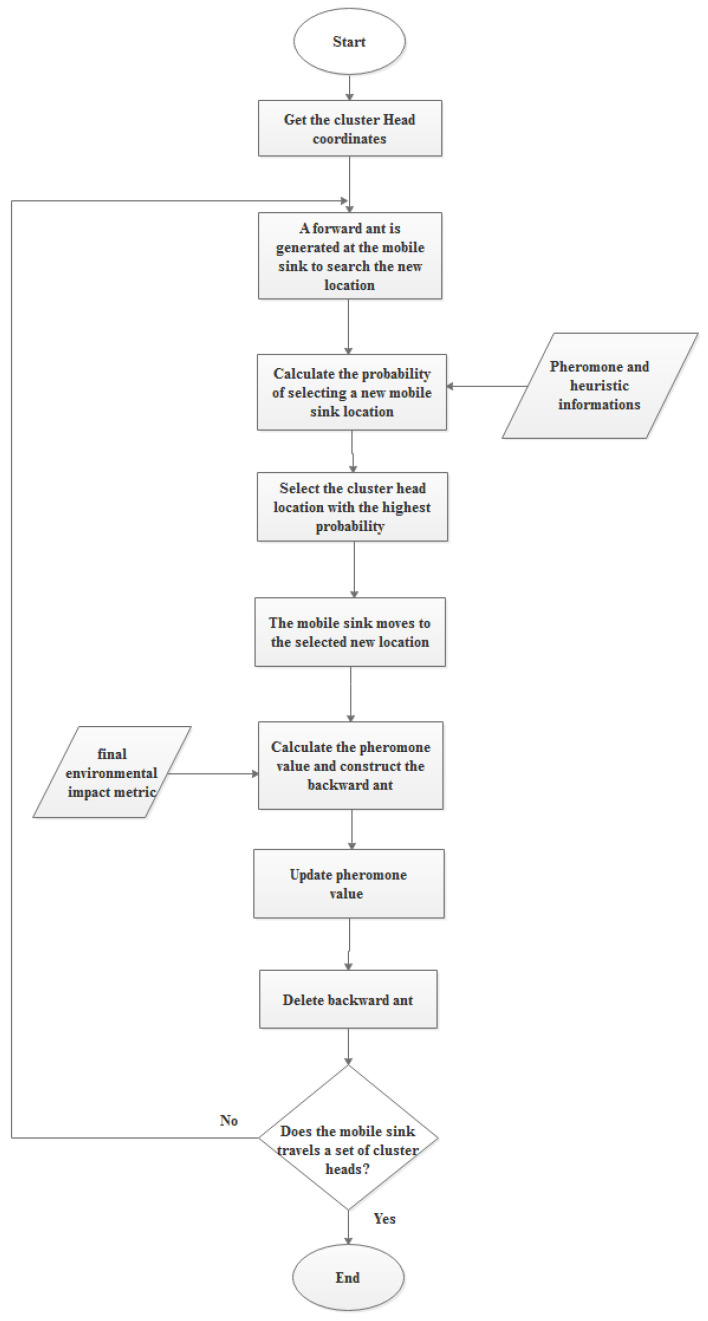
The proposed path planning algorithm flowchart.

In ACO, ants search for a solution to the path planning issue by randomly moving around the cluster head set at each time interval. Each ant *k* begins its tour at each cluster head and constructs connected covers by transitioning between cluster heads in the development graph. Ant *k* applies a probabilistic transition rule at each tour construction step to select which cluster head it will visit next. The probability that ant k, currently at cluster head *u*, will select cluster head *x*, to visit next is given by:

(85) $$P_{ux}^{k}(t)=\frac{{\left[\tau_{ux}^{1}(t)\right]}^{\omega_{1}}{\left[\eta_{ux}^{1}(t)\right]}^{\omega_{2}}{\left[\lambda_{ux}^{1}(t)\right]}^{\omega_{3}}{\left[\beta_{ux}^{1}(t)\right]}^{\omega_{4}}{\left[\gamma_{ux}^{1}(t)\right]}^{\omega_{5}}}{\sum_{l\in FCH^{t}}{\left[\tau_{ul}^{1}(t)\right]}^{\omega_{1}}{\left[\eta_{ul}^{1}(t)\right]}^{\omega_{2}}{\left[\lambda_{ul}^{1}(t)\right]}^{\omega_{3}}{\left[\beta_{ul}^{1}(t)\right]}^{\omega_{4}}{\left[\gamma_{ul}^{1}(t)\right]}^{\omega_{5}}}\;$$

where $\tau_{ux}^{1}(t)$ is the pheromone value between cluster head *u* and *x* at the time *t*, $\eta_{ux}^{1}(t)$, $\lambda_{ux}^{1}(t)$, $\beta_{ux}^{1}(t)$ and $\gamma_{ux}^{1}(t)$ are the heuristic information and $\omega_{1}$, $\omega_{2}$, $\omega_{3}$, $\omega_{4}$, and $\omega_{5}$ are the weight factors that control the pheromone value and the heuristic information parameters, respectively. A flowchart of the proposed cluster head selection approach is shown in Figure 3.

#### 5.1.1. Pheromone Calculation

The pheromone value is updated using the environmental impact metric of the new location since the mobile sink needs to move on a safe path away from the dangerous area. The selection of the new cluster head location with maximum environmental impact is required to lower the influence of environmental factors on the mobile sink movement. At the same time, it helps choose cluster head locations in favorable surroundings and prevent harmful places. Thus, the pheromone increasing density is calculated by the final environmental impact metric of cluster head *x* in zone *i* as follows:

(86) $$\Delta \tau_{ux}^{1}=EIM_{x}^{i}(t).$$

The pheromone trail value is updated as each ant *k*, reaches its new cluster head position according to the following rules:

(87) $$\tau_{ux}^{1}t)=(1-\rho_{1})\tau_{ux}^{1}(t-1)+\rho_{1}\Delta \tau_{ux}^{1}$$

where $\rho_{1}\in \left(0,1\right)$ is the evaporation factor [33].

#### 5.1.2. The Heuristic Information Calculation

In the majority of application scenarios, the mobile sink’s battery acts as its only source of power, and the battery cannot be recharged [11]. As a result, the mobile sink has an energy limit [11]. In fact, the mobile sink movement is the main source of consumed energy and costs. More energy is needed to move when the target cluster head location is near to the movement’s beginning point. Therefore, the closer a mobile sink is to the target location, the more energy efficiently it operates. The remaining energy after the movement is consequently taken into consideration as the following heuristic information to provide minimum movement energy and so improve mobile sink lifespan:

(88) $$\eta_{ux}^{1}(t)=\frac{\left(RE_{MS}\left(t\right)-ME_{\left(u,x\right)}\left(t\right)\right)}{\sum_{l\in FCH^{t}}\left(RE_{MS}\left(t\right)-ME_{\left(u,l\right)}\left(t\right)\right)}.$$

Because the candidate cluster head position has a larger value of $\eta_{ux}^{1}(t)$, the mobile sink will have more residual energy if it is relocated from the cluster head’s location *u* to *x*. Thus, cluster head x has more opportunities to be the next location.

As mobile sink travel may be time-consuming, particularly in large sensor fields, some packets may be missed due to low buffer space. To decrease packet loss and increase network lifetime, a delay-aware method is needed [18]. Hence, the movement delay is considered in the heuristic information as follows:

(89) λux1(t)=1Dux(t)∑l∈FCHt1Dul(t)

where

(90) $$D_{ux}\left(t\right)=\frac{ED_{ux}(t)}{speed_{MS}}.$$

The candidate cluster head location with a greater value of $\lambda_{ux}^{1}(t)$ has more opportunity to be the next location as it requires less time in the movement than other candidates.

To ensure in-time urgent data delivery and avoid excessive routing, the mobile sink should move first to the cluster heads in the high priority zones. As a result, while choosing the next deployment point, the zone priority metric of cluster heads is used as heuristic information, as demonstrated by:

(91) $$\beta_{ux}^{1}(t)=\frac{\zeta +ZPM_{x}^{i}(t)}{\sum_{l\in FCH^{t}}\left(\zeta +ZPM_{l}^{i}(t)\right)}$$

where ζ<1 is constant. The candidate cluster head location with a higher value of $\beta_{ux}^{1}(t)$, meaning that the zone of that cluster head has a higher priority, has a better chance of being the next new location.

It is necessary to reduce the distance between the sink node and urgent cluster heads to increase real-time performance. This reduces the required latency, so important messages arrive on time. The heuristic information considers the average distance between urgent cluster heads as follows:

(92) γux1(t)=1Adx∑l∈FCHt1Adl.

The cluster head location with a higher value of $\gamma_{ux}^{1}(t)$ means that this location will have a lower average distance, and it has more chance of being the next new location of the mobile sink.

### 5.2. Cluster Head Selection Problem

The suggested solution is offered in two stages. When sensor nodes are placed and turned on, the first phase starts. All nodes in a homogeneous network have the same initial energy. Sensor nodes communicate with their neighbors by sending "hello" messages that include node location, ID, and energy remaining. After exchanging messages, nodes generate neighbor tables. The sensor node with the most neighbors is the first cluster head. In a heterogeneous network, the first cluster head is based on node residual energy. Advanced and super nodes are given priority to become cluster heads since they have greater energy.

The ACO algorithm eliminates repetitive rotation and prevents sensor nodes from transmitting a lot of control signals during the second step, cluster head selection.

**Figure 4:**
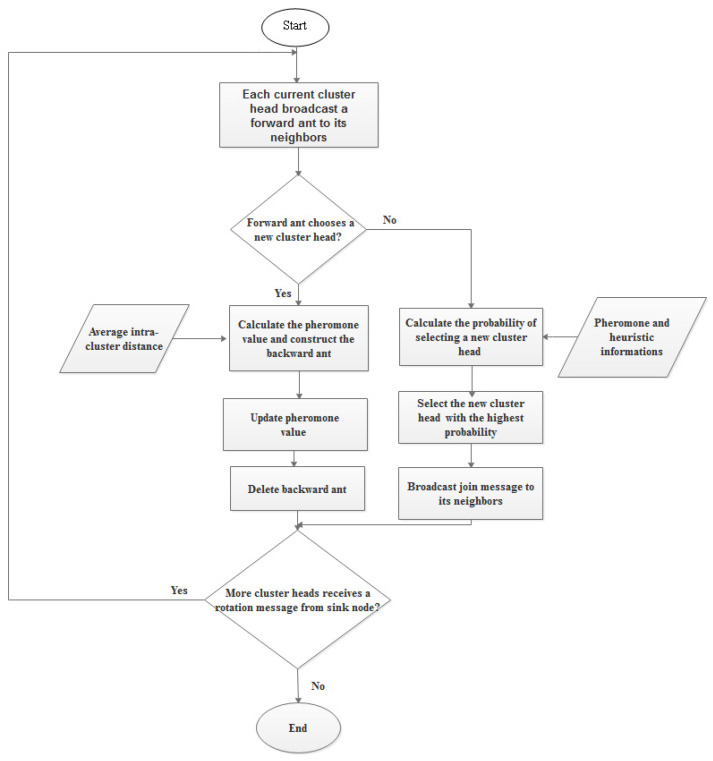
The proposed clustering algorithm flowchart.

During each time interval *t*, the sink node broadcasts an advertisement message to trigger cluster head rotation. Then, each cluster head broadcasts an ant across neighboring nodes to start head rotation. Each neighbor set receives one ant, which ACO designates as the cluster head. The node with the highest probability is chosen to serve as the cluster’s new head after it scans its neighbors. Each of the *k* ants chooses the head node according to a probabilistic rule. Ant k*’s* likelihood of selecting node *x* as the cluster head is:

(93) $$P_{x}^{k}(t)=\frac{{\left[\tau_{x}^{2}(t)\right]}^{\vartheta_{1}}{\left[\eta_{x}^{2}(t)\right]}^{\vartheta_{2}}{\left[\lambda_{x}^{2}(t)\right]}^{\vartheta_{3}}}{\sum_{l\in NM_{x}}{\left[\tau_{l}^{2}(t)\right]}^{\vartheta_{1}}{\left[\eta_{l}^{2}(t)\right]}^{\vartheta_{2}}{\left[\lambda_{l}^{2}(t)\right]}^{\vartheta_{3}}}\;$$

where $\tau_{x}^{2}(t)$ is the pheromone value of node *x* at the time *t*, $\eta_{x}^{2}(t)$ and $\lambda_{x}^{2}(t)$ are the heuristic information, $\vartheta_{1}$, $\vartheta_{2}$, and $\vartheta_{3}$ are the control variables that affect the pheromone value and the heuristic information parameters, respectively. A flowchart of the proposed cluster head selection approach is shown in Figure 4.

#### 5.2.1. Pheromone Calculation

The pheromone value is updated using the average intra-cluster distance to improve the delivery delay:

(94) Δτ2=1ICDx

where

(95) ICDx=∑y∈NMxEDxy|NMx|.

The pheromone trail’s update rule is carried out as follows whenever each ant *k* chooses a new cluster head:

(96) $$\tau_{x}^{2}(t)=(1-\rho_{2})\tau_{x}^{2}(t-1)+\rho_{2}\Delta \tau^{2}$$

where $\rho_{2}\in \left(0,1\right)$ is the evaporation factor [33].

#### 5.2.2. The Heuristic Information Calculation

Since data transmission reliability is a critical concern in resource-constrained WSNs, member nodes must send data packets to cluster heads via a reliable wireless link. However, WSN is typically used in hostile environments. Therefore, packet loss is expected. Packet loss is one of the most critical yet difficult challenges in WSNs since retransmitting missed packets takes more time and energy, reducing in-time delivery and network lifetime. The suggested link quality indicator is utilized as heuristic information to increase reliability, preserve node energy, and promote in-time data delivery as follows:

(97) ηx2(t)=1(1+QMx(t))∑l∈NMx(1(1+QMl(t))).

The node with the highest value has a better chance than the other candidates of being the next cluster head as it has a higher link quality rating.

Due to the data traffic generated by collecting, aggregating, and transferring member data to sink nodes, cluster heads use their remaining energy more quickly than distant nodes. In order to balance node energy consumption and optimize network lifetime, we employ an energy metric, represented by:

(98) $$\lambda_{x}^{2}(t)=\frac{EM_{x}\left(t\right)}{\sum_{l\in NM_{x}}EM_{l}\left(t\right)}.$$

The node with greater $\lambda_{x}^{2}$ has more residual energy and is more likely to be the next cluster head.

### 5.3. Routing Problem

The original ACO method has been updated since it must minimize both time and complexity for the suggested algorithms to be acceptable for real-time applications.

Any urgent data packets should be routed via the cluster head relay nodes until they reach the mobile sink if the cluster head is located in a region that the mobile sink has already visited or has not yet visited, and the cluster head possesses the necessary data. It looks at the cluster heads of its neighbors and unicasts the packet to the best one using the ACO algorithm probability. The packet is then routed via this neighbor, who has chosen the best cluster head among its neighbors, until it reaches the mobile sink. This shows that a route from the cluster head carrying critical data to the mobile sink has been identified, and the data packet arrives there simultaneously. By doing this, the time and complexity of the original ACO method are reduced. It is decided during packet transmission which path the urgent data packet will take.

Two steps make up the suggested solution. If a particular cluster head in the first phase has to communicate urgent data to the mobile sink through the routing process, it sends a forward ant across neighboring cluster head nodes, which serves as a relay node until the mobile sink receives the data. Before choosing the relay nodes, each node must determine which of its cluster head neighbors are allowed to take part in the routing operations. The neighbor nodes that can achieve the necessary delay are the ones that are qualified to be regarded candidate relay nodes and participate in the routing process. The neighbor node is qualified if it can transmit the data packet to the mobile sink in a period of time that is less than or equal to the packet’s remaining deadline. On the other side, we will cooperate with all neighbor nodes if no neighbor node meets such a requirement.

**Figure 5:**
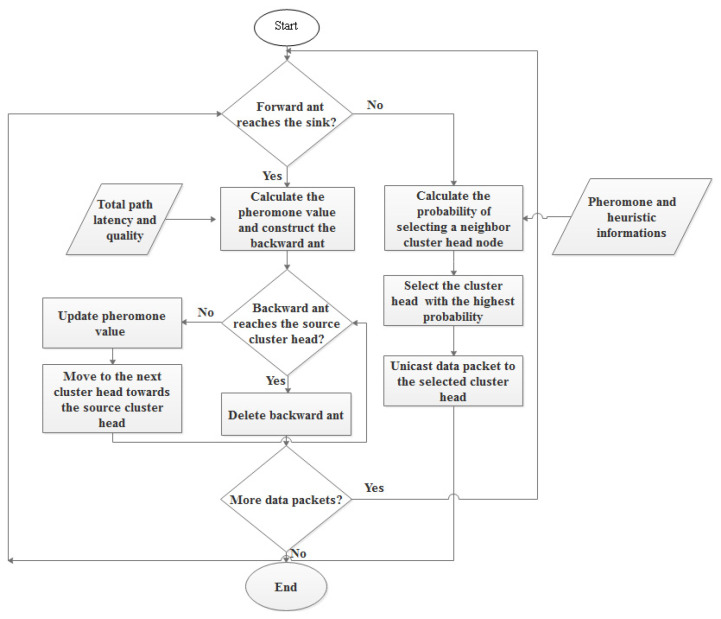
The proposed routing algorithm flowchart.

Second, the forward ant chooses the subsequent hop at each cluster head that serves as a relay node based on a probability. The likelihood of choosing a cluster head neighbor to serve as the next-hop relay node, which is specified by Equation (99), is computed using a number of variables including the relaying latency, environmental effect metric, energy consumption load metric, and connection quality, along with the pheromone value:

(99) $$P_{xy}^{k}(t)=\frac{{\left[\tau_{xy}^{3}(t)\right]}^{\varphi_{1}}{\left[\eta_{xy}^{3}(t)\right]}^{\varphi_{2}}{\left[\lambda_{xy}^{3}(t)\right]}^{\varphi_{3}}{\left[\beta_{xy}^{3}(t)\right]}^{\varphi_{4}}{\left[\gamma_{xy}^{3}(t)\right]}^{\varphi_{5}}}{\sum_{l\in FCHN_{x}}{\left[\tau_{xl}^{3}(t)\right]}^{\varphi_{1}}{\left[\eta_{xl}^{3}(t)\right]}^{\varphi_{2}}{\left[\lambda_{xl}^{3}(t)\right]}^{\varphi_{3}}{\left[\beta_{xl}^{3}(t)\right]}^{\varphi_{4}}{\left[\gamma_{xl}^{3}(t)\right]}^{\varphi_{5}}}\;$$

where $\tau_{xy}^{3}(t)$ is the pheromone value of the link *(x, y)* at the time *t*, $\eta_{xy}^{3}(t)$, $\lambda_{xy}^{3}(t)$, $\beta_{xy}^{3}(t)$, and $\gamma_{xy}^{3}(t)$ are the heuristic information; $\varphi_{1}$, $\varphi_{2}$, $\varphi_{3}$, $\varphi_{4}$, and $\varphi_{5}$ are the control factors that control the pheromone value and the heuristic information parameters, respectively. A flowchart of the proposed routing approach is shown in Figure 5.

#### 5.3.1. Pheromone Calculation

Path quality and latency are used to determine the update pheromone. This improves network reliability and latency. Transmission, propagation, queuing, and processing are end-to-end delays. The processing delay may be removed owing to the sensor nodes speed [15].

The increase in the pheromone density on the path *p* is defined as:

(100) Δτ3=(PRRp)+(IDLdelayp).

Once the forward ant reaches mobile sink, the pheromone update value is computed and transmitted back to its source node via the reverse path as a backward ant. When a cluster head *x* receives a backward ant from its cluster head neighbor *y*, it updates the pheromone concentration according to the following rule:

(101) $$\tau_{xy}^{3}(t)=(1-\rho_{3})\tau_{xy}^{3}(t-1)+\rho_{3}\Delta \tau^{3}$$

where, $\rho_{3}\in (0,1)$ is the evaporation factor [14].

#### 5.3.2. The Heuristic Information Calculation

A final environmental impact metric is taken into account to ensure that the routing protocol is not exposed to environmental impact, which helps select cluster heads in suitable surroundings and bypasses harmful places to a certain extent. Thus, the following heuristic information is calculated by the final environmental impact as follows:

(102) $$\eta_{xy}^{3}(t)=\frac{EIM_{y}^{i}\left(t\right)}{\sum_{l\in FCHN_{x}}EIM_{l}^{i}\left(t\right)}.$$

The candidate cluster head with a higher value of ηij1 has the lower adverse environmental effects on the urgent data packet delivery and is more likely to be selected as the next relay.

To ensure timely delivery of urgent data, the forwarding cluster head with a relaying delay less than the required delay and whose delay is the lowest compared to other candidates should be selected as the next point. Therefore, the relaying delay is considered heuristic information, which is determined by Equation (103) as follows:

(103) $$\lambda_{xy}^{3}(t)=\frac{Rd_{y}(t)}{\sum_{l\in FCHN_{x}}Rd_{l}(t)}.$$

The candidate relay that has a higher value of $\lambda_{xy}^{3}$ offers less delay in the transmission of urgent data and has more chance of being the next relay. Reducing the delivery time will result by selecting the cluster head with the shortest relaying latency at each hop.

The suggested energy consumption load metric is heuristic information that is taken into consideration since energy consumption balance is a critical difficulty in the design of an energy-efficient routing algorithm for WSNs.

(104) $$\beta_{xy}^{3}(t)=\frac{ECL_{y}\left(t\right)}{\sum_{l\in FCHN_{x}}ECL_{y}\left(t\right)}$$

The cluster head with a higher value of $\beta_{xy}^{3}$ has a higher residual energy after sending all its traffic load messages, and thus has a more chance of being selected as the next forwarder.

Some crucial data packets may be lost as a consequence of the lossy links in WSNs, wasting energy and adding to the delay caused by the need to retransmit missing packets. One of the main things that affects end-to-end latency, packet delivery rate, and energy efficiency is packet loss. The lossy feature of wireless connections across it may be described by the *PRR*. As a result, the following is how the path quality is defined. The connection quality is regarded as heuristic data, which is defined as:

(105) $$\gamma_{xy}^{3}(t)=\frac{PRR_{xy}}{\sum_{l\in FCHN_{x}}PRR_{xl}}.$$

The cluster head with a greater value of $\gamma_{xy}^{3}$ has a better link quality and has more opportunity to be chosen as the relay node.

## 6. Performance Evaluations

To assess the effectiveness of our proposal, numerical simulation experiments are carried out in this section. The assessment criteria are presented first. The assessment process is then explained. The simulation results and benchmark comparisons are then shown.

### 6.1. Performance Evaluation Criteria

Four quantitative criteria are considered to evaluate the proposed approach’s performance. These evaluation criteria are explained as follows:

1. Network Lifetime [15] is the time elapsed from the start of the network operation till the first node in the network fails due to battery depletion;
2. Packet delivery ratio (PDR) [15] concerns the number of successful messages received by the sink node to that sent by the source nodes;
3. Deadline miss ratio [15] is the percentage of packets that could not reach the sink node during their deadline;
4. Average end-to-end delay [15] is the average time taken by the data packet to travel from source node to the sink.

### 6.2. Simulation Model

A series of experiments are implemented in the MATLAB tool to validate our proposed algorithms comprehensively. The experiments are carried out in an Intel Core i5 dual-core CPU with a frequency of 2.3 GHz, 4 GB of memory, and a Windows 7 operating system. The simulation environment is composed of a mobile sink and *N* sensor nodes in a squared area of 1000 × 1000 m. After deployment, all sensor nodes are believed to be stationary. The centroid is supposed to be the starting point for the mobile sink to gather data from all cluster heads. The mobile sink should traverse all cluster heads and return to the starting position. In addition, all the later experiments are performed assuming that the network is affected by some environmental events such as wildfire and rainstorms.

To simulate the wildfires, we employed the same radiation model in [13]. A Poisson process is used to generate the data traffic with a mean parameter *λ*. In addition, The WSN lossy links model used in this paper is referred to [34]. Table 4 summarizes the simulation parameters.

We adopted the energy consumption model of [34] to be used in our experiments. This model considers that the total energy consumption by a node mainly includes the energy consumed in transmission and reception of data packets.

### 6.3. Simulation Results

We compare the network lifetime, miss ratio, end-to-end latency, packet delivery ratio, and energy imbalance factor of our approach to those of [18,19,22,24] in order to assess its feasibility and effectiveness. In contrast to the earlier research in [18,19,20,21,22,23,24], all subsequent experiments are carried out with two scenarios since our solution takes the environmental effect into account as one of the key criteria in both the route determination of the mobile sink and the routing mechanism of the urgent data.

1. First scenario: It is considered that environmental statistics are within the normal range; therefore, environmental influences do not affect network performance. It demonstrates the performance of the approaches under normal environmental conditions;
2. Second scenario: It is assumed that the environmental data are outside the normal range. It demonstrates the performance of the approaches under environmental conditions outside the normal range. In the second scenario, all following studies assume the network is impacted by wildfire. Environmental events are considered to occur 400 seconds after network start up.

Additionally, as previously mentioned, none of the earlier studies in [18,19,20,21,22,23,24] take into account the real-time transmission of urgent data packets, while the data from the cluster heads are acquired as the mobile sink approaches each cluster head. As a result, without the usage of a routing protocol, a comparison with the earlier research published in [18,19,22,24] would not be fair. As a result, the suggested routing method is applied with all the earlier research from [18,19,22,24] in all subsequent experiments. The suggested routing algorithm must also be assessed concurrently. As a result, it has to be evaluated against alternative routing techniques. In all subsequent experiments, a different routing method from [28] that is more similar to our suggested approach is taken into account.

As a result, two scenarios are taken into consideration for each situation in order to demonstrate the effectiveness of the proposed approach:

A. Case 1: It exhibits the performance of the techniques using the proposed routing algorithm for urgent data collection;
B. Case 2: It demonstrates the performance of the approaches with the PSO-based routing approach in [28] for urgent data collection.

#### 6.3.1. Network Lifetime Evaluation

In these experiments, the performance of the proposed algorithm is compared to SEA [18], EGTDA [19], and ACO-based techniques in [22], and EARP [24] for the two situations stated above under varied traffic rate.

1. Network lifetime evaluation under the first scenario

**Figure 6:**
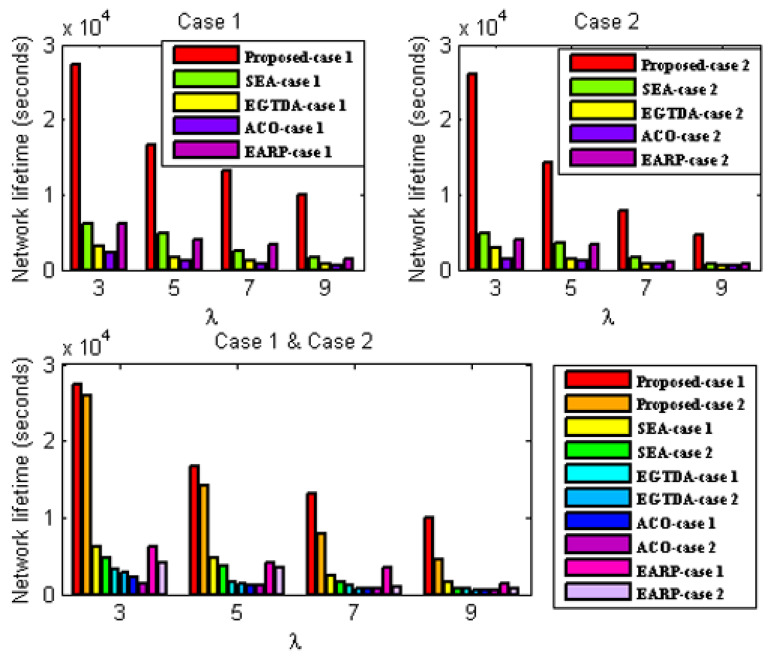
Influence of increasing average traffic rate on network lifetime under the first scenario.

This experiment studies the variation of network lifetime under environmental conditions inside the normal range. The average traffic rate λ varies from three to nine packets per second throughout this experiment. Figure 6 shows the variation of network lifetime to the average traffic rate under the first scenario. As observed in Figure 6, earlier research [18,19,22,24] using the proposed routing algorithm enhanced network lifetime compared to the PSO-based routing technique in [28] for urgent data collection. As in this scenario, the environmental data are assumed to be in the normal range; thus, the corresponding value of the environmental impact metric is 1. This improvement is because the proposed routing algorithm leverages node traffic load and residual energy to balance network energy usage across sensor nodes. It prevents lost links to save energy wasted retransmitting dropped packets. The PSO-dependent routing method in [28] depends on residual energy to balance energy consumption, which is not adequate according to the observations in this work. It attempts to route data along the quickest path, but a lack of knowledge regarding data transmission reliability wastes energy by retransmitting missed packets. The figure illustrates that our idea boosts network lifetime greatly. These findings are due to the following:

The suggested route planning takes into account the zone priority and the energy used when moving the mobile sink, which reduces energy use. Consequently, it significantly increases network lifetime. Messages from sensor nodes situated in high priority zones should be relayed right away to the sink node since they are regarded as urgent data. Due to the excessive routing avoidance of urgent data messages and thus lower energy consumption, including zone priority into the path planning choice causes the mobile sink to collect the urgent data from the high priority zones first. Thus, it increases the network’s lifetime. The lifetime of the mobile sink is extended by accounting for energy loss during mobility. In order to calculate an energy-saving and target-delay-optimal location, it additionally takes into account the average distance between the mobile sink and urgent data cluster heads. The cluster head selection technique balances network energy use among sensor nodes as a result of the suggested energy measure. To cut down on energy wastage from retransmitting failed packets, it also takes link quality into account when choosing a cluster head. Finally, the proposed routing expands the network’s lifetime.

Prior studies [18,19,22,24] do not address zone priority in the route planning approach, which leads to greater energy consumption as a consequence of the routing of urgent data messages since the mobile sink would not go to the high priority zone first. They waste resources by retransmitting failed packets during cluster head selection because they are unaware of the dependability of data transfer from member nodes to cluster heads. The methods employed to control energy use are inadequate to achieve an appropriate energy balance, which shortens the lifetime of the network.

2. Network lifetime evaluation under the second scenario

**Figure 7:**
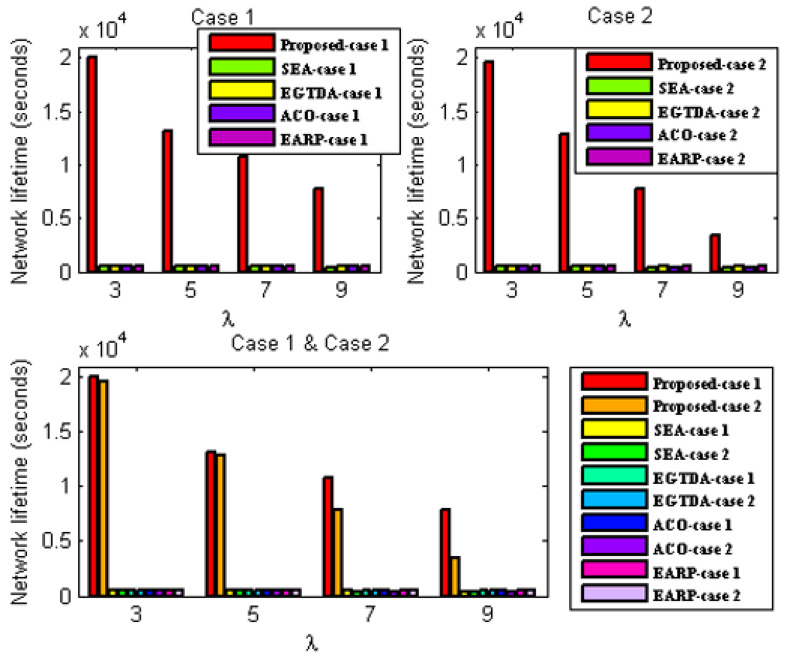
Influence of increasing average traffic rate on network lifetime under the second scenario.

This experiment studies network lifetime under scenario 2. In this experiment, traffic averages three to nine packets per second. Figure 7 displays the second scenario’s network lifetime vs. average traffic rate. The figure indicates that the proposed approach increases network lifetime and traffic rate. As the environmental conditions in this scenario are assumed to be outside the normal range. This is justified as follows:

In addition to the reasons mentioned before, which made the proposed approach outperform the other algorithms in the first scenario, the proposed approach effectively bypasses the danger zones and discovers a safer path for the mobile sink through its modified environmental impact metric. Simultaneously, it tries to reduce the influence of environmental factors on the routing performance of urgent data packets by skipping the risk zone and finding the safest pathways to the mobile sink. In prior research [18,19,22,24], the mobile sink would pass hazardous zones since they do not address environmental influence on network performance. This makes the mobile sink easy to be cut off due to environmental reason, causing the network failure. Therefore, all the previous works in [18,19,22,24] achieve roughly the same lifetime as network failure once the sink node visits a cluster head in a danger zone. In contrast, environmental events occur at 400 seconds from the start of the network operation.

#### 6.3.2. Packets Delivery Ratio (PDR) Evaluation

In this series of tests, the performance of the proposed algorithm is assessed in terms of the packet delivery ratio in comparison to the SEA [18], EGTDA [19], and ACO-based techniques in [22], and EARP [24] for the situations indicated before under varying traffic rates.

1. PDR evaluation under the first scenario

**Figure 8:**
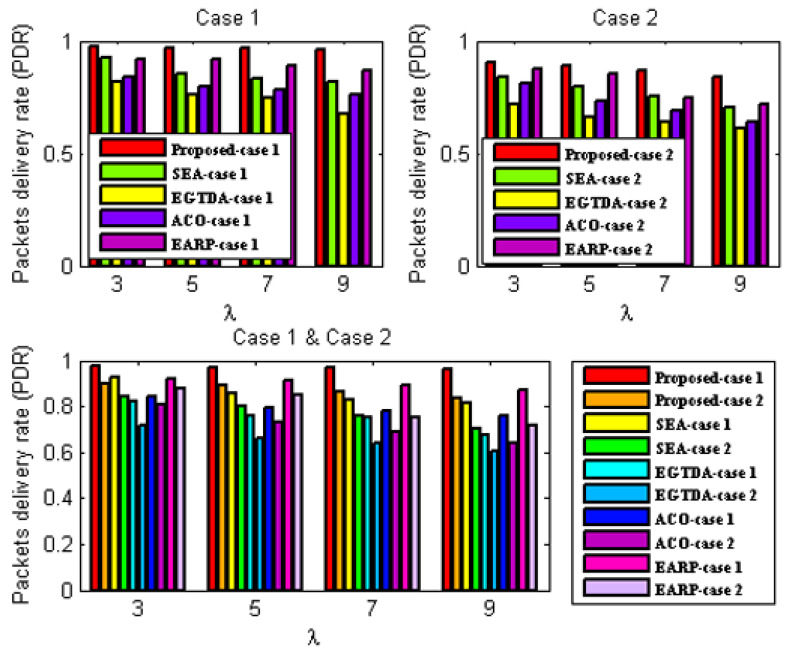
Influence of increasing average traffic rate on packets delivery ratio under the first scenario.

This experiment studies the variation of the network PDR with respect to the average traffic rate under environmental conditions inside the normal range. This experiment started by varying the average traffic rate λ from three to nine packets per second. Figure 8 shows the variation of the network PDR with the average traffic rate under the first scenario. As shown in Figure 8, it is easily observed that the previous works in [18,19,22,24] with the proposed routing algorithm give the highest PDR compared to that with the PSO-based routing approach in [28] for urgent data collection. The environmental data in this scenario are assumed to be in the normal range; thus the corresponding value of the environmental impact metric is one. This enhancement is because the link quality is considered in selecting the next cluster head forwarder and thus delivering data packets more reliably. Regarding the PSO-based routing approach in [28], it does not consider how to avoid the lossy links, leading to more packet loss.

Meanwhile, the proposed approach achieves the highest PDR compared to the others, even while increasing the average traffic rate in the network. This is due to three main reasons: The first reason is that the proposed method attempts to avoid urgent messages routing to the mobile basin by considering the zone priority. Data packets are lost during multi-hop wireless transmission due to the dynamic nature of wireless links and unstable channel conditions. Accordingly, avoiding urgent message routing enhances the PDR of the network; it prevents packets from going to possible unreliable paths. The second reason is that the link quality is integrated into the cluster head selection decision, preventing the forwarding of data packets from member nodes to their cluster heads on unreliable links. The third reason is that the proposed routing algorithm reliably forwards the urgent data packet as described above.

On the contrary, previous works in [18,19,22,24] did not take into account the zone priority; this leads to more packet loss due to the urgent data transmission to the mobile sink through multi-hop routing processes which negatively affects the network throughput. In addition, the reliable data transmission from the member nodes to their cluster heads is not taken into consideration, which increases the packet loss rate and thus diminishes the network throughput.

2. PDR evaluation under the second scenario

**Figure 9:**
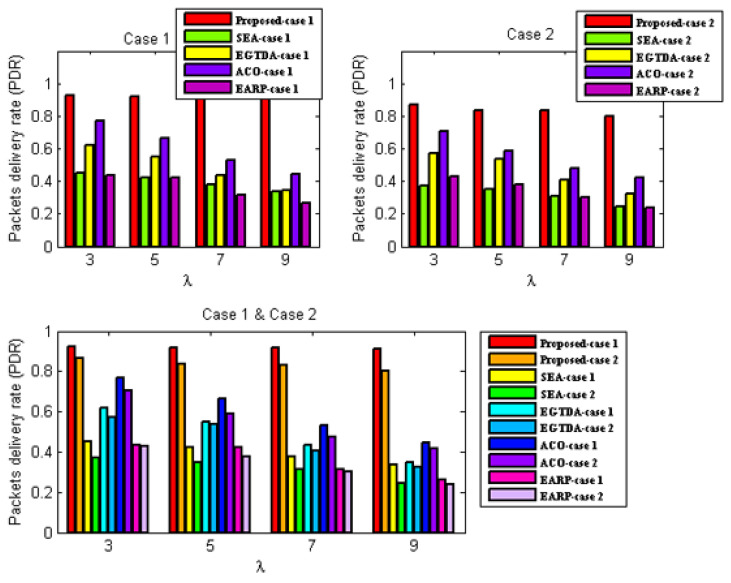
Influence of increasing average traffic rate on packets delivery ratio under the second scenario.

During this experiment, the PDR of the network was investigated using the second scenario, which included varying traffic rates. Under the second case, Figure 9 illustrates the fluctuation in network PDR that occurs with varied traffic rates. For the purpose of putting this variant through its paces, the typical traffic rate λ is varied from three to nine packets per second. When compared to the earlier efforts in [18,19,22,24], this figure demonstrates that the proposed method produces the greatest PDR possible. In light of the fact that it is presumed that the environmental circumstances were outside of the typical range in this situation, the following explanations are provided to justify such results:

The suggested technique outperforms existing algorithms in the first scenario for the reasons indicated above in addition to the fact that it successfully avoids the hazardous zones and finds safer paths for the mobile sink and urgent data transfer via its improved environmental impact measure. The earlier research in [18,19,22,24] does not account for how the environment affects network performance; as a consequence, no action was taken to avoid danger zones, which had a detrimental effect on network dependability since data transmission would stop.

#### 6.3.3. Deadline Miss Ratio Evaluation

In this series of experiments, the performance of the proposed method is assessed in terms of the deadline miss ratio in comparison to the SEA [18], EGTDA [19], and ACO-based techniques in [22], and EARP [24] for the situations indicated above under varying traffic rates. For the sake of evaluating such variations, it is assumed that the urgent data messages have varying deadlines which are determined by the priority of their respective zones. That is to say, the time limit for the transmission of urgent data messages originating from the region with the greatest priority is the shortest, and vice versa. The deadlines range from 100 milliseconds to 700 milliseconds.

1. Deadline miss ratio evaluation under the first scenario

**Figure 10:**
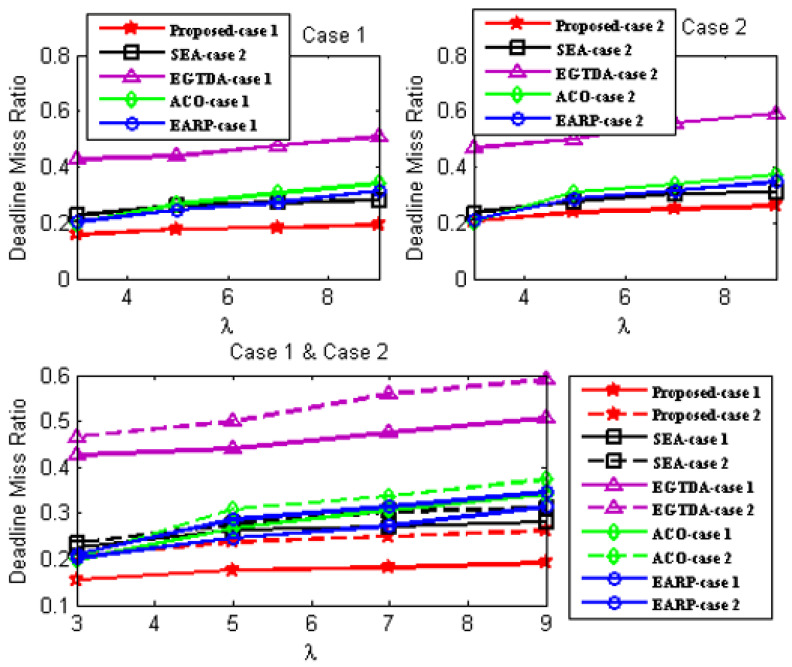
Influence of increasing average traffic rate on deadline miss ratio under the first scenario.

This experiment compares deadline miss ratio with average traffic rate. The simulation experiment is launched to examine this variance by raising the average traffic rate λ from three to nine packets per second. Figure 10 compares deadline miss ratios with various traffic rates. As shown in the figure, the proposed routing method in [18,19,22,24] for urgent data gathering produced a lower deadline miss percentage than the PSO-based routing technique in [28]. The proposed routing method updates the desired delay of packets in each hop and picks cluster heads that can deliver the urgent data packet within its deadline. The relaying delay is then considered while picking the next forwarder from these cluster heads. It evaluates link quality to reduce retransmission latency. Such parameters reduce packet miss ratio.

Real-time data transmission is not included in [28]’s PSO-based routing algorithm, hence constrained latency requirements are not met. Moreover, packets in such an algorithm cannot avoid unreliable paths, causing many lost packets and increasing retransmission latency. So, deadline misses rise.

However, according to the results in Figure 10, it is obvious that the proposed approach achieves the minimum deadline miss ratio compared to other works. The reasons behind such results are justified as follows:

The proposed route planning considers the average distance between the mobile sink and urgent data cluster heads. This reduces the desired delay, which means that the urgent messages would reach the sink node within their deadlines, which in turn reduces the percentage of times that a deadline was missed. According to the information presented earlier, the priority of the zone is considered when determining the path that will be taken. The mobile sink as a result gathers the urgent data from the high priority zones first, resulting in an excessive avoidance of the routing of urgent data messages. It reduces delivery delays as a consequence, which in turn reduces the proportion of deadlines missed. Additionally, the movement delay is considered since the mobile sink’s travel time may be prolonged, especially in large sensor fields, and some packets may be lost owing to the sensor nodes’ limited buffer capacity. As a result, including such a setting results in a decrease in the delay brought on by the retransmission of lost packets. The proposed technique also takes into consideration the average distance between nodes inside the cluster when choosing cluster heads. By reducing end-to-end latency, this also lowers the proportion of deadlines that are missed. Additionally, the cluster heads take connection quality into account while making decisions, which reduces the number of packets lost and the possibility that they will need to be resent. As a result, there are fewer missed deadlines, which improves the timely transmission of important data messages. Last but not least, as was previously stated, the suggested approach achieves the lowest percentage of missed deadlines.

On the other hand, the preceding studies described in [18,19,22,24] did not take into account real-time communications, in which tight latency requirements need to be satisfied. Additionally, they neglected to account for zone priority, which causes delivery delays that are exacerbated by the multi-hop routing of crucial data connections. In turn, this causes a rise in the number of deadlines missed. A considerable percentage of packets are lost as a consequence of their failure to consider the dependability of the data transfer from the member nodes to the cluster heads, which also increases the delay that arises from packet retransmission; in turn, the proportion of deadlines missed will increase.

2. Deadline miss ratio evaluation under the second scenario

**Figure 11:**
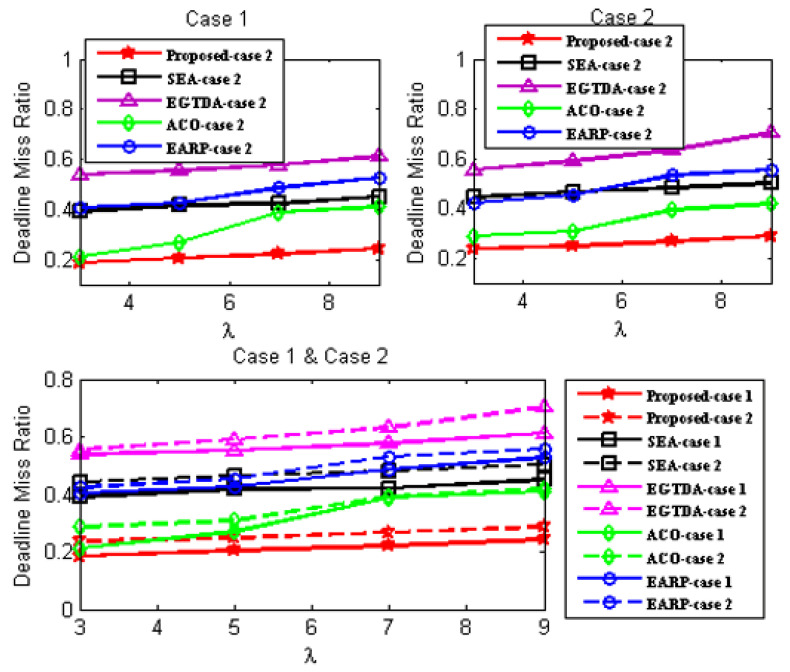
Influence of increasing average traffic rate on deadline miss ratio under the second scenario.

For the second scenario, the effectiveness of the suggested technique is evaluated in this experiment and contrasted to earlier research [18,19,22,24]. The experiment involves sending between three and nine packets per second. Figure 11 shows how the proportion of missed deadlines changes in relation to traffic volume. This figure demonstrates that the suggested method increases average network traffic rate while delivering the lowest deadline miss ratio among the available solutions. This is because the suggested method uses a modified environmental effect measure to find safer routes away from risk areas for the mobile sink and urgent data transfer. Built-in data paths are less susceptible to environmental interruption. As a result, there is less packet loss and retransmission during packet transit, which lowers delivery delay. This demonstrates the superiority of the suggested approach. The reasons the suggested method performed better than other algorithms in the first case are revealed in the remainder.

Meanwhile, the previous works performed in [18,19,22,24] cannot make prompt reactions to the environmental changes; therefore, they cannot provide sustainable message delivery services under harsh environmental conditions. This increases the packet loss rate, resulting in more delivery delays due to the retransmission of lost packets and increasing the deadline miss ratio.

#### 6.3.4. Average End-to-End Delay Evaluation

A further series of experiments are carried out in this part with the purpose of evaluating the suggested technique in terms of end-to-end latency. Comparisons are made between the proposed method and SEA [18], EGTDA [19], and ACO-based techniques in [22], and EARP [24] for the situations indicated above at varying traffic rates.

1. Average end-to-end delay evaluation under the first scenario

**Figure 12:**
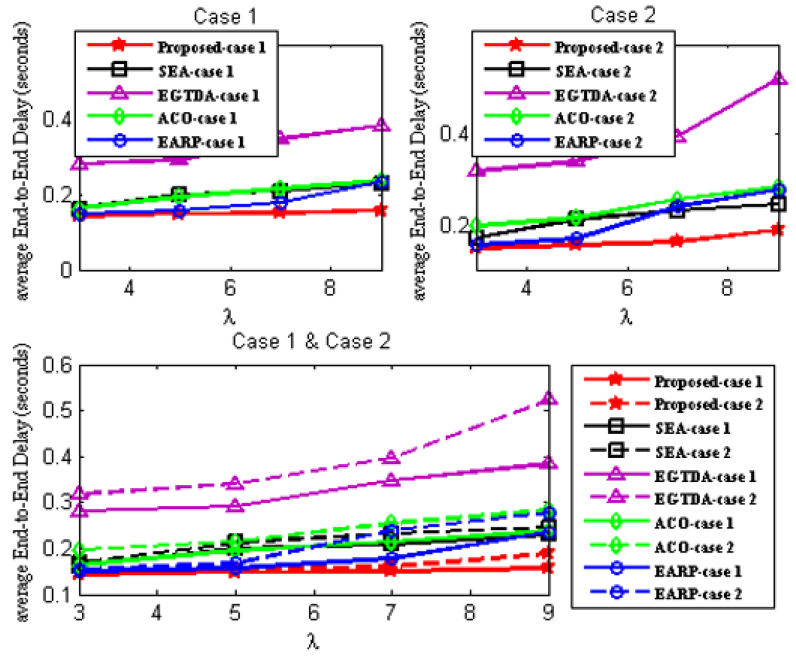
Influence of increasing average traffic rate on average end-to-end delay for the first scenario.

The primary purpose of this simulation experiment is to analyze average end-to-end latency with average traffic rate in the first scenario. This experiment varied traffic from three to nine packets per second. Figure 12 depicts end-to-end delay changes with traffic volume. The previous studies [18,19,22,24], using the suggested routing algorithm, had the lowest end-to-end latency compared to [28]’s PSO-based routing technique for urgent data collecting. It picks forwarding nodes that can transmit the packet on a more stable connection, reducing packet loss and retransmission, which reduces delivery latency. In addition, it picks the cluster heads that can transmit the urgent data packet on time. The relaying delay is used to pick the next forwarder from these cluster heads, resulting in quick packet delivery and reduced delivery latency. In the scenario described in [28], using the PSO-based routing technique, packets are unable to bypass the lossy links, which results in an increase in the end-to-end latency as a result of the retransmission of lost packets. Because it does not enable real-time communications, the transmission of data will be delayed as a result.

As the suggested route design takes zone priority into account, the proposed method has the shortest end-to-end latency. As was just said, the delivery time may be reduced by increasing the average distance that separates the mobile sink from the cluster heads that have urgent data messages and increasing the movement delay. In addition, the suggested method for selecting cluster heads takes into account the average distance travelled inside the cluster as well as the quality of the links between clusters. This helps reduce the end-to-end latency, as was discussed before. In conclusion—again, as was discussed before—the suggested route results in the least amount of delay from beginning to finish. In the case of the previous works in [18,19,22,24], they did not consider the real-time communications and the zone priority, which leads to more delivery delays. Moreover, they do not consider the reliable data transmission from the member nodes to their cluster heads, which caused an increase in the end-to-end delay due to the retransmission of lost packets.

2. Average end-to-end delay evaluation under the second scenario

**Figure 13:**
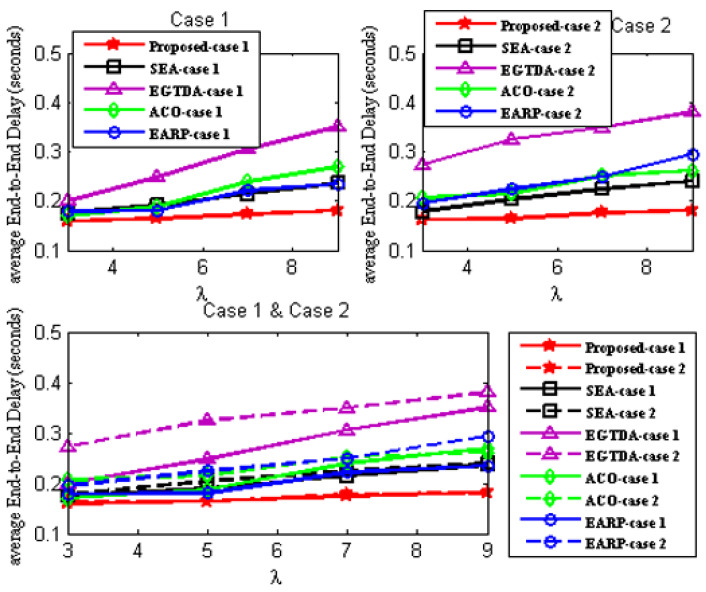
Influence of average traffic rate on average end-to-end delay for the second scenario.

This simulation experiment studies how average end-to-end latency varies with traffic rate in the second scenario. This simulation experiment varied from three to nine packets per second. Figure 13 demonstrates how end-to-end latency varies with traffic volume. This figure shows that the recommended strategy reduces end-to-end latency. The suggested solution outperformed other algorithms in the first scenario for the reasons indicated above. In addition, the proposed approach attempts to construct data routes less likely to be cut off due to environmental reasons, leading to delivery of data packets on more reliable links and thus less delivery delay.

## 7. Conclusions

This paper introduced several novel techniques for WSNs including path planning, an efficient clustering algorithm, an efficient real-time routing algorithm, and novel path planning. The routing algorithm considered real-time messages, sensors energy, and urgent messages while the path planning considered the environmental effect on the monitored areas. The method for clustering took into account the sensor nodes’ energy, the quality of the wireless links, and the distance parameter, which represented the average distance between clusters. The path planning utilized the areas’ priority, environmental parameters, and urgent messages. The suggested routing method is environment and energy aware, taking into account load balancing, environmental data, network quality, latency, and hop count. These algorithms serve as the foundation for WSN operations. Based on the comprehensive experiments employed in this research, we draw the conclusion that the suggested framework is effective and has surpassed current methods. The limitation of the proposed algorithms is the time complexity; they take into account more realistic parameters than the comparison algorithms and this will take more computation time. In the future of this research, it may be extended to take into account multiple sink nodes. The research may also be expanded to include other uses, such as scheduling and mobility for drones.

## Figures and Tables

**Table 1:** A comparative summary of the algorithms mentioned above and the proposed ones.

Paper ID	Path Planning							Cluster Head Selection		
	Parameters							Parameters		
	Moving Cost	Travelling Delay	Traveling Distance	Cluster Head Energy	Areas (Zones) Priority	Environmental Impact	Real Time Delivery	Residual Energy	Distance	Reliability
[18]	√	√	√	×	×	×	×	√	×	×
[19]	×	×	√	√	×	×	×	√	×	×
[20]	×	√	×	×	×	×	×	√	√	×
[21]	×	×	×	√	×	×	×	√	√	×
[22]	×	×	√	×	×	×	×	√	×	×
[23]	×	×	√	×	×	×	×	√	√	×
[24]	×	×	√	×	×	×	×	√	√	×
Proposed Solution	√	√	√	×	√	√	√	√	√	√

**Table 2:** A comparative summary of the two algorithms mentioned earlier [27,28] and the proposed one.

Paper ID	Cluster-Based Routing					
	Parameters					
	Distance	Residual Energy	Traffic Load	Reliability	Environmental Impact	Real Time Delivery
[27]	√	√	×	×	×	×
[28]	√	√	×	×	×	×
Proposed Solution	√	√	√	√	√	√

**Table 3:** The problems model notations.

Given Parameters	
Notation	Description
*V*	Set of the monitored field sensor nodes.
*MS*	The mobile sink.
*T*	The time horizon.
*Z*	The set of all zones in the monitored field.
$speed_{MS}$	The mobile sink movement speed, *MS*.
$CH^{t}$	The cluster heads set in the monitored field at time *t*, $t\in T,CH^{t}\in V$.
$FCH^{t}$	The final set of cluster heads that have an environmental impact metric larger than threshold value in the monitored field at time t, $t\in T,FCH^{t}\in CH^{t},CH^{t}\in V$.
$CHU^{t}$	The cluster heads set that have urgent messages at time t, $t\in T,CHU^{t}\in FCH^{t},FCH^{t}\in CH^{t},and\;CH^{t}\in V$.
$UM_{s}^{t}$	The set of all urgent messages collected from the members of the cluster head s at time t, $\forall s\in CHU^{t},CHU^{t}\in CH^{t},CH^{t}\in V,and\;t\in T.$
*p*	The mobile sink moving path, S at time t, which consists of a set of all vertices from *v_i_* to *v_n_*, $v_{i},v_{n}\in CH^{t},CH^{t}\in V,t\in T.$
$C_{ux}\left(t\right)$	The movement cost from point *u* to *x*, $u,x\in CH^{t},CH^{t}\in V,t\in T$
$D_{ux}\left(t\right)$	The movement delay from point *u* to *x*, $u,x\in CH^{t},CH^{t}\in V,t\in T$
$ZED_{i}^{f}$	The zone *i’s* environmental data with environmental factor *f*.
$EI_{i}^{f}$	Zone *i* environmental impact metric for single environmental factor *f*.
$MEI_{i}$	Environmental impact metric of zone *i* for multiple environmental factors.
$NEI_{i}$	The neighboring environmental impact metric of zone *i*.
$FEI_{i}$	The final environmental impact metric of zone *i*.
$EIM_{x}^{i}(t)$	The final environmental impact metric of the cluster head *x* in a zone *i* at time *t*, $x\in CH^{t},CH^{t}\in V,i\in Z,t\in T$.
$EI_{th}$	The environmental impact threshold.
$K_{ij}$	The attenuation coefficient between zone *i* and *j*.
$ED_{ij}$	the distance between zones i and j centers where *i* ≠ *j*.
$ED_{\left(x,MS\right)}(t)$	The distance between both the cluster head x and sink node MS at time *t*, $x\in CH^{t},CH^{t}\in V,t\in T$.
$Hc_{\left(x,MS\right)}(t)$	The hop count from the cluster head *x* to sink node *MS* at time *t*, $x\in CH^{t},CH^{t}\in V,t\in T$.
$ME_{\left(u,x\right)}\left(t\right)$	The energy needed to transfer mobile sink *s* from cluster head *u* to *x* at time *t*, $x\in CH^{t},CH^{t}\in V,t\in T$.
$ZP_{i}(t)$	The zone priority of zone *i* at time *t*, i∈Z,t∈T.
$ZPM_{x}^{i}(t)$	The zone priority metric of the cluster head *x* in zone *i* at time *t,* $x\in CH^{t},CH^{t}\in V,i\in Z,t\in T$.
*IDL*	The initial deadline is defined at each source node by the application.
*deadline*	The packet deadline is updated at each hop.
$Dd_{x}$	The required delivery delay for the message-based cluster head *x*, $x\in CHU^{t},CHU^{t}\in V$.
$Ad_{x}$	Cluster head average distance x, $x\in CHU^{t},CHU^{t}\in V$.
$ICD_{x}$	Is the intra-cluster distance of node *x*, x∈V.
$PRR_{xy}$	The packet reception ratio for the link *(x,y)*, (x,y)∈V and x≠y.
$PLR_{xy}$	The packet loss ratio for the link *(x,y)*, (x,y)∈V and x≠y.
$D_{xy}$	The delay for the link *(x,y)*. (x,y)∈V and x≠y.
$RE_{x}(t)$	Residual energy of node *x* at time *t*, $x\in CH^{t},CH^{t}\in V,t\in T$
$IE_{x}$	Sensor node *x* initial energy, $x\in CH^{t},CH^{t}\in V$
$HE_{xy}$	Energy required for Single hop transmission from *x* to *y*, $x,y\in CH^{t},CH^{t}\in V$
$ECL_{y}(t)$	Energy consumption load function for each cluster head *y* at time *t*, $y\in NEB_{x},NEB_{x}\in CH^{t},CH^{t}\in V,t\in T$
$NL_{y}(t)$	The load of each cluster head *x* at time *t*, $y\in NEB_{x},NEB_{x}\in CH^{t},CH^{t}\in V,t\in T$
$TM_{y}\left(t\right)$	The total number of messages at cluster head *y*, $y\in CH^{t},CH^{t}\in V,t\in T$
$P_{y}^{t}$	The set of all candidate paths between any pair *(y, MS)*, $\forall y\in CH^{t},CH^{t}\in V,and\;t\in T$
$NM_{x}$	The neighbor set of node x that is not covered by any cluster head, $x,NM_{x}\in V.$
$NEB_{x}$	The neighbor set of node *x*, $x,NEB_{x}\in V.$
$CCHN_{x}$	The cluster head neighbor set of cluster head x, $x,CCHN_{x}\in CH^{t},CH^{t}\in V,and\;t\in T.$
$FCHN_{x}$	The final cluster head neighbor set of cluster head *x*, $x,FCHN_{x}\in CH^{t},CH^{t}\in V,and\;t\in T.$
Indicator Parameter	
$\alpha_{y}^{p}$	1 if cluster head y is on path *p* to the mobile sink and 0 otherwise, $\forall y\in FCHN_{x},p\in P_{y}^{t},FCHN_{x}\in CH^{t},CH^{t}\in V,and\;t\in T.$
Decision Variables	
$\xi_{\left(i,i+1\right)}$	1 if the sink node *MS* moves from point *i* to *i*+1 and 0 otherwise, $i,i+1\in CH^{t},CH^{t}\in V$.
$X_{ux}^{t}$	1 if the mobile sink moves from cluster head *u* to cluster head *x* at time intervals *t* and 0 otherwise, $\forall t\in T,u,\forall x,u\in CH^{t}\;{and\;CH}^{t}\in V.$
$d_{xy}^{su}$	1 if the cluster head *x* uses the link (*x*, *y*) to transmit urgent message *u* through it to sink node and 0 otherwise, $\forall u\in UM_{s}^{t},\forall s\in CHU^{t},\;x,y\in CH^{t},CH^{t},CHU^{t}\in V,and\;t\in T.$
$\omega_{xy}^{su}$	1 if the cluster head *x* uses cluster head *y* to relay urgent message *u* of the source node s and 0 otherwise, $\forall u\in UM_{s}^{t},\forall s\in CHU^{t},\;x,y\in CH^{t},CH^{t},CHU^{t}\in V,and\;t\in T$
$r_{x}$	1 if the cluster head *x* has a final environmental metric greater than the threshold value and 0 otherwise, $\forall x\in CH^{t},{\;\mathrm{and}\;CH}^{t}\in V.$
$E_{n}$	1 if the difference between cluster head x’s environmental metric and cluster head n’s is less than zero and 0 otherwise. $\forall x\in CH^{t},n\in CH^{t}-\{x\},\;i,j\;\in {Z\;\mathrm{and}\;CH}^{t}\in V.$
$g_{\upsilon }$	1 if the difference between the zone priority metric of cluster head *x’s* in zone *i*, and cluster head *v’s* in zone *j* is less than 0, 0 otherwise. $\forall x\in CH^{t},n\in CH^{t}-\{x\},\;i,j\;\in {Z\;\mathrm{and}\;CH}^{t}\in V.$
$h_{k}$	1 if the difference between the average distance metric value of cluster head *x* in zone *i* and cluster head *k* in zone *j* is less than zero and 0 otherwise, $\forall x\in CH^{t},n\in CH^{t}-\{x\},\;i,j\;\in {Z\;\mathrm{and}\;CH}^{t}\in V.$
$I_{x}$	1 if the cluster head *x* has the highest environmental metric value compared to that of other cluster heads and 0 otherwise, $\forall x\in CH^{t}{\;\mathrm{and}\;CH}^{t}\in V.$
$P_{x}$	1 if the cluster head *x* has the highest zone priority metric value compared to that of other cluster heads and 0 otherwise, $\forall x\in CH^{t}{\;\mathrm{and}\;CH}^{t}\in V.$
$m_{x}$	1 if the cluster head *x* has the minimum average distance metric value compared to that of other cluster heads and 0 otherwise, $\forall x\in CH^{t}{\;\mathrm{and}\;CH}^{t}\in V.$
$\psi_{x}$	1 if the node *x* is elected as a cluster head and 0 otherwise, x∈V
$\delta_{x}^{t}$	1 if the node *x* is selected as a cluster head at time interval *t* and 0 otherwise, ∀t∈T,∀x∈V.
$\varepsilon_{f}$	1 if the difference between the quality metric values of node *x* and node *f* is less than zero and 0 otherwise, ∀x∈V,f∈V−{x}
$\mu_{b}$	1 if the difference between the energy metric values node *x* and node *b* is less than zero and 0 otherwise, ∀x∈V,b∈V−{x}
$q_{x}$	1 if the node *x* has the minimum quality metric value compared to that of other nodes and 0 otherwise, ∀x∈V.
$R_{x}$	1 if the node *x* has the highest energy metric value compared to that of other nodes and 0 otherwise, ∀x∈V.
$c_{y}$	1 if the cluster head *y* has relaying delay less than or equal to the desired delay and 0 otherwise, $\forall y\in CCHN_{x},CCHN_{x}\in CH^{t},CH^{t}\in V,and\;t\in T.$
$o_{y}$	1 if cluster head *y* can reach sink node and 0 otherwise, $\forall y\in FCHN_{x},FCHN_{x}\in CH^{t},CH^{t}\in V,and\;t\in T.$
$de_{y}$	1 if the cluster head *y* has the minimum value of the relaying delay compared with other cluster heads and 0 otherwise, $\forall y\in FCHN_{x},FCHN_{x}\in CH^{t},CH^{t}\in V,and\;t\in T.$
$a_{l}$	1 if the difference between the relaying delay value of cluster heads l and *y* is less than zero and 0 otherwise, $\forall y\in FCHN_{x},l\in FCHN_{x}-\{y\},FCHN_{x}\in CH^{t},CH^{t}\in V,t\in T.$
$e_{y}$	1 if the cluster head *y* has the highest value obtained from energy load function value compared to that of other cluster heads and 0 otherwise, $\forall y\in FCHN_{x},FCHN_{x}\in CH^{t},CH^{t}\in V,and\;t\in T.$
$b_{\upsilon }$	1 if the difference between the obtained values from energy load function of cluster head *y* and *ν* is less than zero and 0 otherwise, $\forall y\in FCHN_{x},\upsilon \in FCHN_{x}-\{y\},FCHN_{x}\in CH^{t},CH^{t}\in V,t\in T.$
$v_{n}$	1 if the difference between the environmental metric value of cluster head y and n is less than zero and 0 otherwise, $\forall y\in FCHN_{x},n\in FCHN_{x}-\{y\},FCHN_{x}\in CH^{t},CH^{t}\in V,t\in T.$
$N_{y}$	1 if the cluster head *y* has the maximum environmental metric value compared to that of other cluster heads and 0 otherwise, $\forall y\in FCHN_{x},,FCHN_{x}\in CH^{t},CH^{t}\in V,t\in T.$

**Table 4:** Simulation environment parameters.

Parameters	Values
Node deployment strategy	Uniformly random
Number of sensor nodes	200
Maximum retransmissions number	4
Packet size	50 bytes
Buffer size	128 bytes
Frequency Path loss exponent	868 MHz 3
Transmission power Initial energy of nodes Noise floor	0 dBm 125 mJ −115 dBm
Maximum radio range	150 m
Data rate	20 Kbps
Shadow fading variance	3
Reference distance	1 m
Event area (Wildfire)	Circles with a radius from 5 m and 50 m
Normal range for temperature	[0, 50] °C
Normal range for relative humidity	[30%, 80%]
Extreme range (temperature)	[−10, 100] °C
Extreme range (relative humidity)	[0%, 100%]

## Data Availability

Not applicable.
